# Investigating Glioblastoma Response to Hypoxia

**DOI:** 10.3390/biomedicines8090310

**Published:** 2020-08-27

**Authors:** Agathe L. Chédeville, Anbarasu Lourdusamy, Ana Rita Monteiro, Richard Hill, Patricia A. Madureira

**Affiliations:** 1 Institute of Biomedical and Biomolecular Sciences, University of Portsmouth, Portsmouth PO1 2DT, UK; agathe.chedeville@etu.univ-rouen.fr (A.L.C.); rita.monteiro95@gmail.com (A.R.M.); drrjhill@gmail.com (R.H.); 2 Children’s Brain Tumour Research Centre, School of Medicine, University of Nottingham, Biodiscovery Institute, Nottingham NG7 2RD, UK; anbarasu.lourdusamy@nottingham.ac.uk; 3 Centre for Biomedical Research, University of Algarve, Gambelas Campus, room 2.22, 8005-099 Faro, Portugal

**Keywords:** glioblastoma, hypoxia, glycolysis, invasion, autophagy, tumor angiogenesis

## Abstract

Glioblastoma (GB) is the most common and deadly type of primary malignant brain tumor with an average patient survival of only 15–17 months. GBs typically have hypoxic regions associated with aggressiveness and chemoresistance. Using patient derived GB cells, we characterized how GB responds to hypoxia. We noted a hypoxia-dependent glycolytic switch characterized by the up-regulation of HK2, PFKFB3, PFKFB4, LDHA, PDK1, *SLC2A1*/GLUT-1, *CA9*/CAIX, and *SLC16A3*/MCT-4. Moreover, many proangiogenic genes and proteins, including VEGFA, VEGFC, VEGFD, *PGF*/PlGF, ADM, ANGPTL4, and *SERPINE1/*PAI-1 were up-regulated during hypoxia. We detected the hypoxic induction of invasion proteins, including the plasminogen receptor, S100A10, and the urokinase plasminogen activator receptor, uPAR. Furthermore, we observed a hypoxia-dependent up-regulation of the autophagy genes, *BNIP-3* and *DDIT4* and of the multi-functional protein, NDRG1 associated with GB chemoresistance; and down-regulation of *EGR1* and *TFRC* (Graphical abstract). Analysis of GB patient cohorts’ revealed differential expression of these genes in patient samples (except *SLC16A3*) compared to non-neoplastic brain tissue. High expression of *SLC2A1*, *LDHA*, *PDK1*, *PFKFB4*, *HK2*, *VEGFA*, *SERPINE1*, *TFRC*, and *ADM* was associated with significantly lower overall survival. Together these data provide important information regarding GB response to hypoxia which could support the development of more effective treatments for GB patients.

## 1. Introduction

Glioblastoma (GB), classified by the World Health Organization (WHO) as a grade IV astrocytoma is the most common and deadly type of primary malignant brain tumor, with a patient median survival of only 15–17 months following diagnosis [1,2,3].

The standard of treatment for GB patients is the “Stupp protocol” which comprises tumor resection surgery (if possible based on MRI imaging), followed by concomitant radiotherapy and chemotherapy with temozolomide [4]. While radiotherapy alone has been shown to significantly increase overall survival, subgroup analysis determined that clinical response to temozolomide was limited to those tumors containing *O6-methylguanine DNA methyltransferase* (*MGMT*) promoter methylation [1,5]. Critically, irrespective of *MGMT* methylation, almost all GB patients develop resistance to therapy and succumb to the disease [6].

Despite the development of more effective therapies for many types of cancers, GB treatment has not changed since 2005 [4]. New targets and treatments against this devastating disease are therefore urgently required. To this end it is essential to better understand the complex biology of GBs and their response(s) to the tumor microenvironment.

A main characteristic of GB is the presence of hypoxic cores (O_2_ partial pressure less than 10 mmHg) that are associated with both tumor aggressiveness and chemoresistance. GB pathological features include necrotic foci with surrounding cellular pseudopalisades and microvascular hyperplasia, which are associated with rapid growth and invasion [5]. Research suggests that pseudopalisades are created by GB cells migrating away from hypoxic regions and creating invasive fronts. Microvascular hyperplasia, is an exacerbated form of angiogenesis (formation of new blood vessels from preexisting vessels) that occurs in response to the secretion of proangiogenic factors by the GB cells that form the pseudopalisades [5]. The excessive Vascular Endothelial Growth Factor A (VEGFA) production observed in GB favors the hyper-proliferation and recruitment of endothelial cells in detriment of pericytes that cover and support the blood vessels [5]. This results in the formation of weak, permeable vessels that frequently collapse creating hypoxic foci within the GB.

The hypoxic response is mainly regulated by the transcription factors Hypoxia Inducible Factors (HIFs), HIF-1 and HIF-2. The regulation of the alpha subunit of HIF (HIF-𝛼) is mediated by the action of Prolyl Hydroxylases (PHDs) that in the presence of normal levels of oxygen (normoxia) are able to hydroxylate HIF-𝛼 at two prolyl residues. This modification allows the protein Von Hippel-Lindau (pVHL) to bind to HIF-α and to recruit E3-ubiquitin ligases which target HIF-𝛼 for proteasomal degradation [6]. Hypoxia inhibits PHDs and enables HIF-𝛼 accumulation in the cell. HIF-𝛼 then translocates into the nucleus where it binds to the constitutively expressed HIF-1β (also known as Aryl Hydrocarbon Receptor Nuclear Translocator, ARNT) subunit and cofactors such as CBP/p300 inducing the transcription of hundreds of genes involved in the regulation of angiogenesis, glycolysis, epithelial-to-mesenchymal transition, proliferation, invasion, and inflammation [6].

Invasion is a main challenge to total GB resection. The plasminogen system and the Matrix Metallo-Proteases (MMPs), in particular MMP-2 and MMP-9, constitute two main systems involved in extra-cellular matrix (ECM) degradation, invasion and metastasis in many cancers [7,8]. The plasminogen system is constituted by the plasminogen activators, tissue-type Plasminogen Activator (tPA) and urokinase Plasminogen Activator (uPA); their inhibitors, Plasminogen Activators Inhibitors −1 and −2 (PAI-1, PAI-2); the receptor for uPA, uPAR; and by cellular plasminogen receptors. The plasminogen activators, tPA and uPA cleave the Arg561-Val562 peptide bond of the inactive zymogen (pro-enzyme) plasminogen, generating the disulfide bond-linked 2-chain serine protease, plasmin [7,8]. Endothelial cells secrete tPA, whereas uPA is produced by many different types of cells including endothelial, inflammatory and cancer cells. Increasing evidence has shown that the cellular receptors for plasminogen play a major role in cancer progression [7,8]. Plasminogen binding to the cells significantly increases the rate of plasmin activation because it promotes the co-localization of plasminogen with its activators, tPA and uPA. Moreover, certain plasminogen receptors can bind to tPA directly further stimulating plasmin formation. Cellular receptor-mediated binding of plasminogen also promotes its proteolytic activity by protecting the newly generated plasmin from inactivation by α2-antiplasmin. The Annexin A2 (ANXA2)-S100A10 heterotetramer is an important plasminogen receptor, associated with tumor invasion and metastasis [7,8]. This receptor is constituted by two molecules of ANXA2 bound together by an S100A10 dimer. ANXA2 has phospholipid binding motifs that are responsible for anchoring this plasminogen receptor to the cell membrane, whereas the S100A10 moiety possesses C-terminal lysine residues that can bind to both tPA and plasminogen [7,9].

The endopeptidases, MMPs also play an important role in tumor invasion mainly via ECM degradation. MMPs can be grouped into collagenases, gelatinases, matrilysins, stromelysins, glycosylphosphatidylinositol-anchored MMPs, transmembrane type I and II MMPs, and other MMPs, based on substrate specificity and structural organization. MMPs are synthesized as inactive zymogens (pro-MMPs) and their activation involves the proteolytic cleavage either by trypsin, other MMPs, plasmin, by allosteric activation, or by chemical modification elicited for example by reactive oxygen species (ROS); followed by the autocatalytic removal of the pro-peptide [10].

Considering the key role that hypoxia plays in GB progression and chemoresistance, the characterization of GB response to this microenvironmental stress is central to the identification of important molecular markers and therapeutic targets.

Here, we utilized several patient derived GB cell lines to investigate how they respond to hypoxia (1%O_2_). We observed a hypoxia-dependent glycolytic switch characterized by the up-regulation of HK2, PFKFB3, PFKFB4, LDHA, PDK1, *SLC2A1*/GLUT-1, *CA9*/CA IX, and *SLC16A3*/MCT-4 genes and proteins in all GB cells investigated. We noted the hypoxic up-regulation of many proangiogenic genes and proteins, namely VEGFA, VEGFC, VEGFD, *PGF*/PlGF, *ADM*, *ANGPTL4*, and *SERPINE1/*PAI-1. We detected a hypoxic induction of invasion proteins, including the plasminogen receptor, S100A10, and the urokinase plasminogen activator receptor, uPAR. Moreover, we observed a hypoxia-dependent up-regulation of the autophagy genes, *BNIP-3* and *DDIT4* and of the multi-functional protein, NDRG1, associated with GB chemoresistance. Our study further revealed the down-regulation of *EGR1* and *TFRC* during hypoxia. Analysis of GB patient data indicated the differential expression of these genes (except *SLC16A3*) in GB samples compared to non-neoplastic brain tissue. Importantly, high expression of *SLC2A1*, *LDHA*, *PDK1*, *PFKFB4*, *HK2*, *VEGFA*, *SERPINE1*, and *ADM* in GB was associated with significantly worse overall survival. Together, these data highlight the response of GB cells to hypoxia, providing important information that could support the development of new and effective therapies for these patients in the future.

## 2. Experimental Section

### 2.1. Cell Lines and Cell Culture

Human adult glioblastoma biopsy-derived primary cell cultures: UP-007, UP-029, SEBTA-003, and SEBTA-023 were obtained from patients from Kings College Hospital, London, under ethics permission (REC reference number: 11/SC/0048, 29 August 2018). Establishment of the aforementioned cell lines is detailed in [11,12]. Details of the cell lines derived from primary adult glioblastomas are summarized on Table S1. U87 and MDA-MB-231 cell lines were obtained from ATCC, Manassas, VA, USA. Cells were grown in DMEM (HyClone™, Logan, UT, USA) supplemented with 10% fetal bovine serum (FBS) (Merck, Dorset, UK), and 20 mM L-Glutamine (Merck, Dorset, UK). Human adult non-neoplastic astrocytes, SC-1800 were obtained from ScienCell Research Laboratories, Carlsbad, CA, USA. SC-1800 cells were grown in AGM Astrocyte Growth Medium BulletKit (LONZA, Slough, UK). Cells were maintained in a humidified incubator with 21% O_2_ (normoxia) or 1% O_2_ (hypoxia) at 37 °C with 5% CO_2_. Cells were tested for mycoplasma contamination regularly. All cells used in these experiments tested negative for mycoplasma contamination.

### 2.2. Antibodies

Antibodies are listed on Table S2.

### 2.3. Hypoxia Signaling Pathway RT2 PCR Profiler Array

10^6^ cells were grown in 100 mm plates for 24 h. After what cells were subjected to either normoxia (21% O_2_), or hypoxia for 6 h or 48 h (1% O_2_). RNA was then extracted using the NZY Total RNA Isolation kit (Nzytech, Lisbon, Portugal) according to the manufacturer´s instructions. RNA quality was analyzed using an RNA 6000 Nano Kit chip assay (Agilent, Stockport, Cheshire, UK) in a 2100 Bioanalyzer (Agilent, Stockport, Cheshire, UK) to ensure RNA extracts had an integrity index value greater than 9.0. 2 µg of each RNA was used for cDNA synthesis using the RT2 first strand kit (QIAGEN, Manchester, UK) according to the manufacturer’s instructions. The Hypoxia Signaling Pathway RT2 Profiler PCR Array; ref: PAHS-032Z (QIAGEN, Manchester, UK) was performed according to the manufacturer’s instructions in a LightCycler 96 instrument (Roche, London, UK).

### 2.4. RT-qPCR

RNA was isolated using the NZY Total RNA Isolation kit (ref: MB13402, Nzytech, Lisbon, Portugal) according to the manufacturer’s instructions. The gene expression was measured by RT-qPCR using the One-step NZYSpeedy RT-qPCR Green kit (ref: MB34602, Nzytech, Lisbon, Portugal) according to the manufacturer´s instructions in a LightCycler 96 instrument (Roche, London, UK). Gene expression levels were normalized to *RPLP0* mRNA using the 2^−ΔΔCT^ method [13]. Error bars represent the Standard Deviations obtained from the median value of at least three independent experiments, each performed in triplicate. The primers used for RT-qPCR are described on Table S3.

### 2.5. Western Blotting

Cells were treated as described in the results section and extracts were prepared with lysis buffer (5 mM EDTA, 50 mM Tris pH 7.5, 120 mM NaCl, 1% NP-40, 10 mM NaF, 5 mM NaVO_4_, protease inhibitors), incubated on ice for 10 min and centrifuged at 12,000× *g* for 15 min at 4 °C. Supernatants containing total cell extracts were transferred to fresh Eppendorf tubes and stored at −80° until needed. For Western blotting, 20 μg of each cell lysate was subjected to SDS-PAGE, transferred onto a nitrocellulose membrane (Biotrace NT, Pall, Portsmouth, UK), incubated with appropriate antibodies and visualized using a Licor Odyssey CLx Imaging system (LI-COR Biosciences, Cambridge, UK).

### 2.6. Statistical Analysis

The statistical significance was evaluated using two-tailed Student’s t-test. Gene expression at different hypoxia time-points (for each time-point) were compared with the expression of the same gene under normoxic conditions. The *p* values were calculated from at least three independent experiments (N ≥ 3).

Changes were only considered significant if there was at least a 2-fold change during hypoxia relative to normoxic conditions (cut-off = 2).

### 2.7. Human GB Transcriptome Data

The raw microarray gene expression data (GeneChip™ Human Genome U133 Plus 2.0 Array) were downloaded from the ArrayExpress database (E-MTAB-3073). Expression intensity values were calculated at probeset level using the robust multi-array average (RMA) method. Probesets that are “absent” (present/absent call using MAS5) in all samples were filtered out from the analysis. Expression values were mapped from probeset to unique gene and the probeset with the highest mean expression value was selected when multiple probesets were mapped to the same gene. To identify differentially expressed genes, linear models were fitted with Bioconductor’s Limma package, which uses a moderated t-statistic based on empirical Bayesian method. The *p* values were adjusted for multiple comparisons using the Benjamini and Hochberg’s false discovery (FDR) procedure. The Kaplan–Meier (KM) product limit method was used to estimate the empirical survival probabilities. Patient samples were classified as high or low (group) based on the median expression value for selected genes and survival differences among groups were compared by the log rank test using the Rpackage “survival”.

RNA sequencing data (Fragments Per Kilobase of transcript per Million (FPKM) mapped reads) was downloaded from the Gene Expression Omnibus (GEO) database. GEO is a public functional genomics data repository, which supports various types of high-throughput experimental data submission. The GEO series (GSE59612) contain the FPKM data for 17 non-neoplastic brain tissue samples, 39 contrast-enhancing GB core samples and 36 non-enhancing GB invasive margin samples. The FPKM data was converted to Transcripts Per Kilobase Million (TPM) and transformed to log_2_ (TPM + 1). The Bioconductor’s Limma was used to test the normalized data for differential expression of non-neoplastic brain vs. contrast-enhancing GB core samples, and non-neoplastic brain vs. non-enhancing GB invasive margin samples. The *p* values were adjusted for multiple comparisons using the Benjamini and Hochberg’s false discovery (FDR) procedure.

## 3. Results

### 3.1. Expression of Hypoxia Related Genes in Glioblastoma Cells

**Figure 1:**
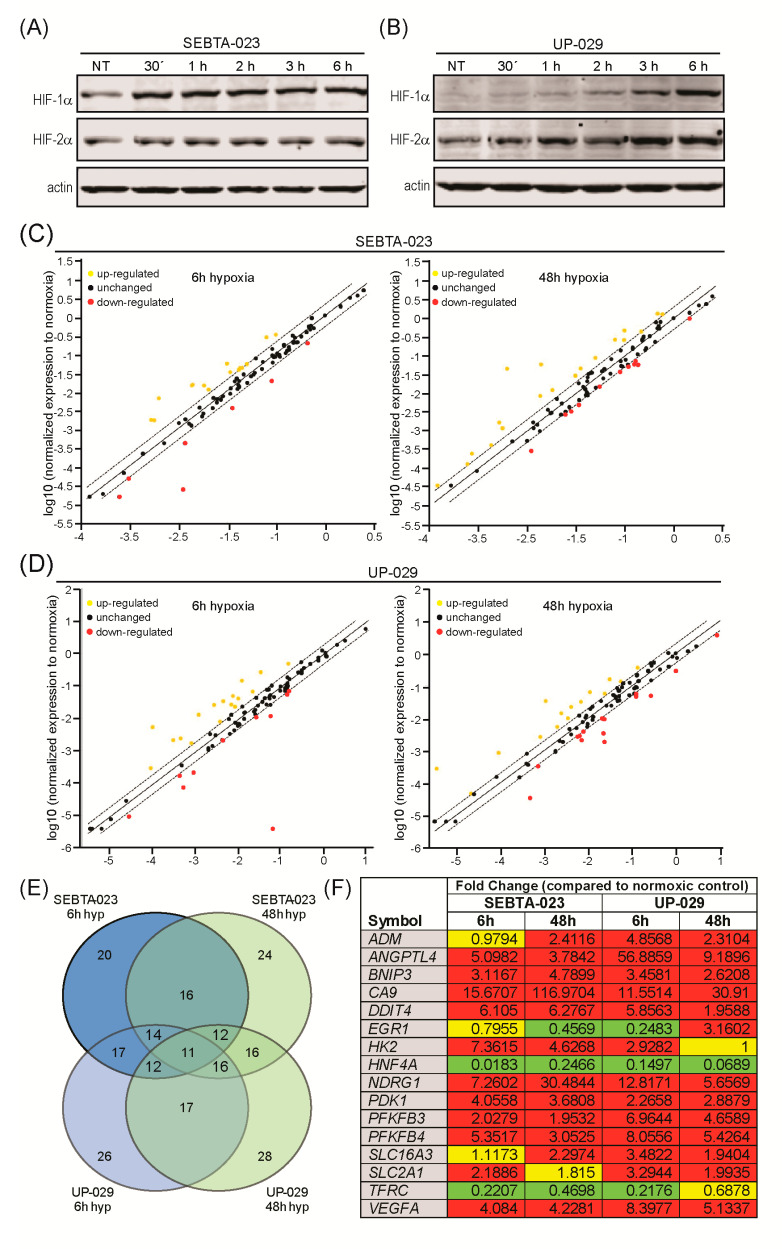
Analysis of hypoxia related genes in SEBTA-023 and UP-029 cell lines. SEBTA-023 and UP-029 cells were either incubated under normoxic (NT) conditions (21% O_2_) or under hypoxia (1% O_2_) for the times indicated. (**A**) SEBTA-023 or (**B**) UP-029 cell lysates were prepared and 20 µg of each protein extract was subjected to SDS-PAGE and analyzed by Western blotting with the antibodies indicated. (**C**,**D**) RNA extraction was performed using the NZY Total RNA Isolation kit (Nzytech, Lisbon, Portugal). A panel of 86 ROS dependent genes was analyzed using the Hypoxia Signaling Pathway RT^2^ Profiler PCR Array (QIAGEN, Manchester, UK). (**C**) Scatter plots for gene expression in hypoxia vs. normoxia in SEBTA-023 cells; (**D**) Scatter plots for gene expression in hypoxia vs. normoxia in UP-029 cells; (**E**) Venn diagram representing differential gene expression in hypoxia vs. normoxia in SEBTA-023 and UP-029 cells. (**F**) Table containing the genes that were differentially expressed in both SEBTA-023 and UP-029 cells throughout the hypoxia time-course. Yellow highlights unchanged genes, red highlights over-expressed genes and green highlights down-regulated genes in hypoxia compared to normoxic conditions.

To investigate the response of GB cells to hypoxia we first subjected SEBTA-023 and UP-029 patient derived GB cells to normoxia (21% O_2_), or to 6 h and 48 h of hypoxia (1% O_2_). Hypoxia dependent gene expression was analyzed using the Hypoxia Signaling Pathway RT2 Profiler PCR Array (QIAGEN, Manchester, UK). To validate the presence of a hypoxic environment we conducted Western blot analysis for HIF-1α and HIF-2α proteins (Figure 1A,B). We observed the accumulation of HIF-1α and HIF-2α in a hypoxia time-dependent manner in both GB lines analyzed, indicative of a hypoxic environment. Gene expression analysis in SEBTA-023 cells revealed the differential expression of 20 genes at 6 h, and 24 genes at 48 h post-hypoxia incubation compared to normoxic control cells; 16 genes were conserved in both hypoxia time-points (Figure 1C,E and Table S4). In UP-029 cells, we observed the differential expression of 26 genes at 6 h, and 28 genes at 48 h post-hypoxia incubation; 17 genes were conserved in both hypoxia time-points (Figure 1D,E and Table S4). Moreover, 24 differentially expressed genes were common to both UP-029 and SEBTA-023 cells in at least 1 hypoxia time-point (6 h or 48 h) (Table S4), and 16 genes were differentially expressed in both cell lines throughout the hypoxia time-course compared to their respective normoxic control cells (Figure 1F and Table S4). These genes were the following, up-regulation of Adrenomedullin (ADM); Angiopoietin Like-4 (ANGPTL4); BCL2/adenovirus E1B 19kDa Interacting Protein 3 (BNIP3); Carbonic Anhydrase 9 (CA9); DNA-Damage-Inducible Transcript 4 (DDIT4); Hexokinase 2 (HK2); N-myc Downstream Regulated 1 (NDRG1); Pyruvate Dehydrogenase Kinase, isozyme 1 (PDK1); 6-Phosphofructo-2-Kinase/Fructose-2,6-Biphosphatase 3 and 4 (PFKFB3; PFKFB4); Solute Carrier family 16, member 3 (SLC16A3); Solute Carrier family 2 (facilitated glucose transporter), member 1 (SLC2A1) and Vascular Endothelial Growth Factor A (VEGFA); down-regulation of Hepatocyte Nuclear Factor 4, alpha (HNF4A) and Transferrin Receptor (TFRC). Differential expression of Early Growth Response 1 (EGR1).

**Figure 2:**
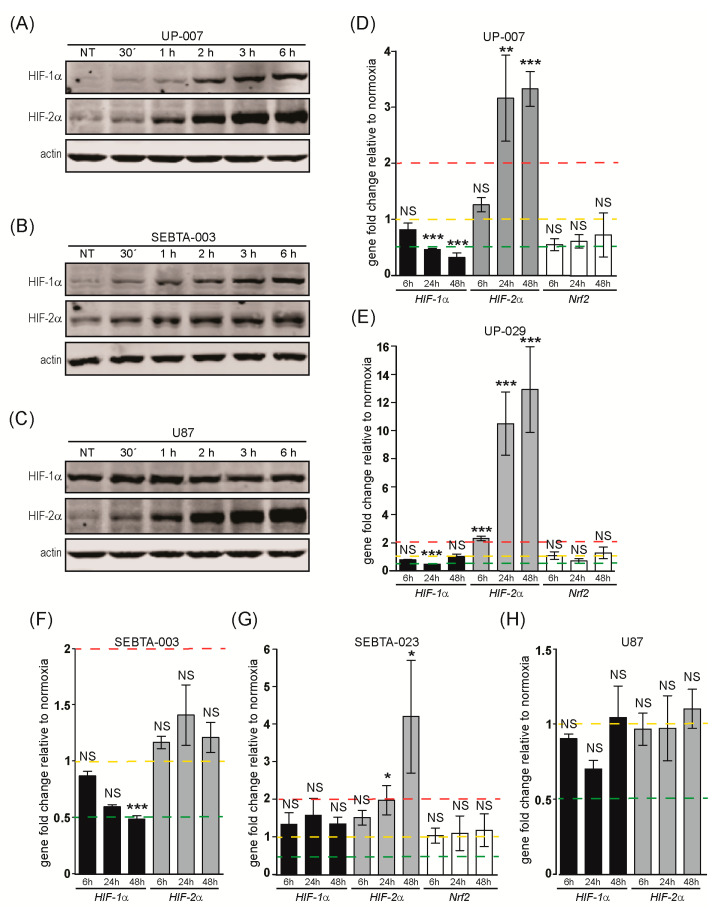
Analysis of transcription factor genes and proteins in hypoxic Glioblastoma (GB) cells. (**A**) UP-007; (**B**) SEBTA-003; or (**C**) U87 cells were incubated under normoxia (21% O_2_) or hypoxia (1% O_2_) for the times indicated. Cells were lysed and 20 µg of each protein extract was subjected to SDS-PAGE and analyzed by Western blotting with the antibodies indicated. (**D**) UP-007; (**E**) UP-029; (**F**) SEBTA-003; (**G**) SEBTA-023; or (**H**) U87 cells were incubated under normoxia (21% O_2_) or hypoxia (1% O_2_) for the times indicated. RNA extraction was performed and gene expression was determined by RT-qPCR. Gene expression levels were normalized to RPLP0 mRNA using the 2^−ΔΔCT^ method [13]. Error bars represent the Standard Deviations obtained from the median value of at least three independent experiments, each performed in triplicate. Statistical analysis was evaluated using two-tailed Student’s *t*-test, comparing each hypoxia time-point to the respective normoxic control. In every case a *p* value of less than 0.05 (*), less than 0.01 (**), and 0.001 (***) was considered statistically significant.

We next performed RT-qPCR analysis for the differentially expressed genes to confirm our RT2 Profiler PCR array findings and to broaden these data to more hypoxia time-points and to a wider number of GB cell lines. In addition, we included a number of related genes that were not present in the Hypoxia Signaling Pathway RT2 Profiler PCR array. These were the invasion genes: S100A10/p11, MMP-2, and uPAR; the proangiogenic genes: VEGFC and VEGFD; and the transcription factors: HIF-2α and Nrf2. For these studies, we used the following patient derived GB cells: UP-007, UP-029, SEBTA-003, SEBTA-023, and the commercially available, and widely studied, U87 cell line. We confirmed a normoxic/hypoxic environment by Western blot analysis for HIF-1α and HIF-2α protein expression (Figure 1A,B and Figure 2A–C). These data showed accumulation of both HIF-1α and HIF-2α in a hypoxia time-dependent manner in all cell lines analyzed, with the exception of U87 cells. In this cell line, we only observed a hypoxia-dependent increase in HIF-2α (Figure 2C). HIF-1α protein levels were already high/stable under normoxic conditions in U87 cells and did not change during hypoxia (Figure 2C).

#### 3.1.1. Transcription Factors Gene Expression Analysis

Analysis of HIF-1α and HIF-2α transcription revealed that HIF-1α mRNA levels were unchanged in SEBTA-023 and U87 cells during hypoxia, transiently down-regulated at 24 h of hypoxia in UP-029 cells and down-regulated in a time-dependent manner in hypoxic UP-007 and SEBTA-003 cells compared to matched normoxic control cells (Figure 2D–H and Table S5). HIF-2α transcription increased significantly in UP-007, UP-029 and SEBTA-023 cells in a hypoxia time-dependent manner, whereas no change in HIF-2α expression was observed in SEBTA-003 or U87 cells throughout the course of hypoxia (Figure 2D–H and Table S5).

It is well established that hypoxia leads to the enhanced production of intracellular ROS [6]. For this reason, we analyzed the expression of Nrf2, which constitutes the main transcription factor regulating the cellular oxidative stress response. Nrf2 transcription did not change in any of the GB cells analyzed during hypoxia (Figure 2D,E,G and Table S5). Similarly, we did not observe any hypoxia-dependent changes in Nrf2 protein levels in our GB cell lines (data not shown).

To determine the endogenous transcription levels (and abundancy) of our genes of interest, we compared the expression of each gene relative to Ribosomal Protein Lateral stalk subunit P0 (RPLP0) housekeeping gene in normoxic GB cells. RPLP0 was also used for RT-qPCR data normalization throughout the transcriptional studies. These data revealed that the relative expression of HIF-1α in UP-007 and SEBTA-003 cells was approximately 0.5–0.6 compared to RPLP0 on a scale of 0–1, corresponding to a −1.7 to −2 fold, and about 0.2 (−5 fold) in UP-029, SEBTA-023 and U87 cells (Figure S1A–E). Compared to HIF-1α the relative expression of HIF-2α was lower, 0.1-0.2 (−5 to −10 fold) in comparison to RPLP0 in SEBTA-003 and U87 cells and 0.05 (−20 fold) compared to RPLP0 in UP-007, UP-029, and SEBTA-023 cells (Figure S1A–E). Interestingly, we only observed a hypoxia-dependent up-regulation of HIF-2α transcription in the GB cells that showed lower endogenous levels of this gene relative to RPLP0 (Figure 2D,E,G and Figure S1A,B,D).

#### 3.1.2. Expression Analysis of Glycolysis Related Genes

**Figure 3:**
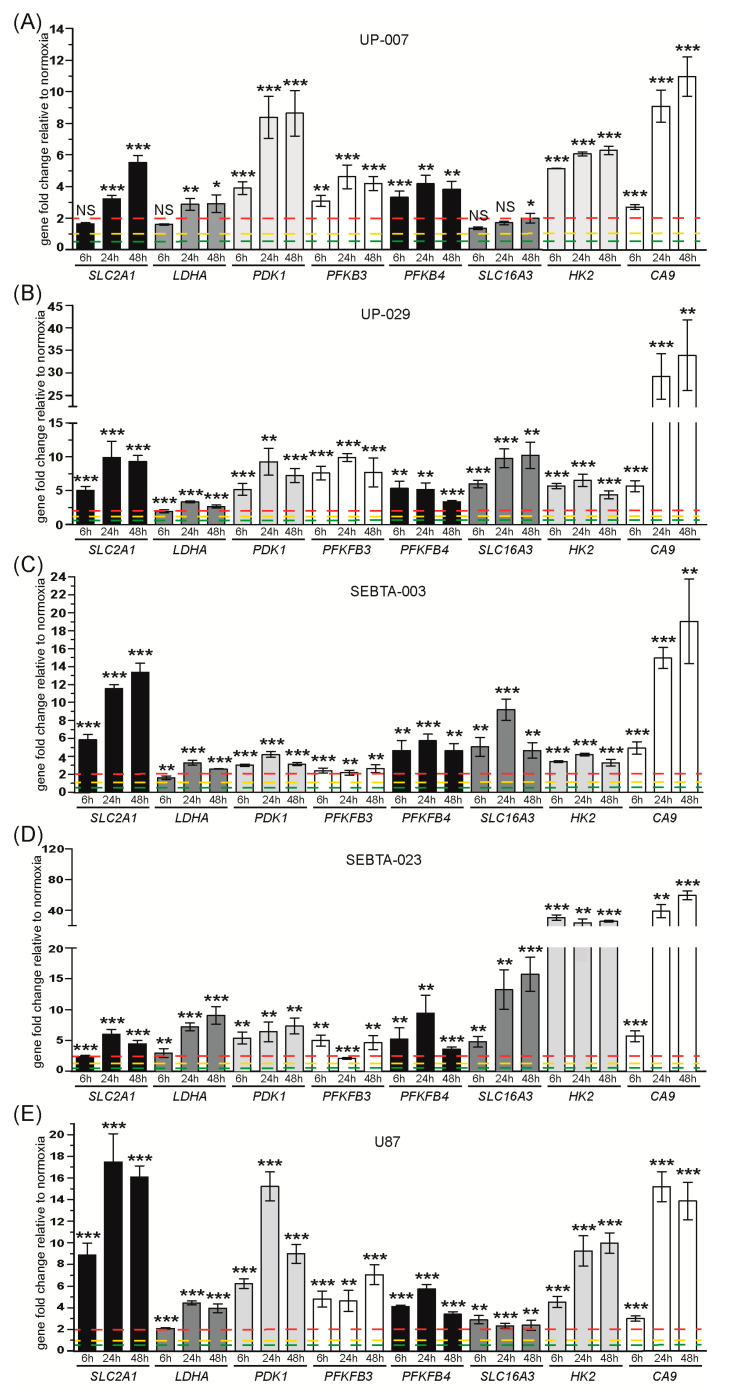
Analysis of glycolysis related genes in hypoxic GB cells. (**A**) UP-007; (**B**) UP-029; (**C**) SEBTA-003; (**D**) SEBTA-023; or (**E**) U87 cells were incubated under normoxia (21% O_2_) or hypoxia (1% O_2_) for the times indicated. RNA extraction was performed and gene expression was determined by RT-qPCR. Gene expression levels were normalized to RPLP0 mRNA using the 2^−ΔΔCT^ method [13]. Error bars represent the Standard Deviations obtained from the median value of at least three independent experiments, performed in triplicate. Statistical analysis was done using two-tailed Student’s t-test, comparing each hypoxia time-point to the respective normoxic control. In every case a *p* value of less than 0.05 (*), less than 0.01 (**), and 0.001 (***) was considered statistically significant.

We next analyzed the expression of glycolysis related genes (Figure 3 and Table S5). These data showed a significant 5–10 fold up-regulation of the genes that encode the glycolytic enzymes: HK2, PFKFB3, PFKFB4, and PDK1 throughout the hypoxia time-course in all GB cell lines investigated; with the exception of PFKFB3 expression in SEBTA-003 cells which was only 2 fold during hypoxia and HK2 expression in SEBTA-023 cells which was up-regulated by approximately 35 fold during hypoxia compared to normoxic control cells. LDHA transcription was 3–5 fold up-regulated in UP-007, UP-029, SEBTA-003, and U87 cells during hypoxia; and 5–10 fold in hypoxic SEBTA-023 cells. We observed a 5–10 fold hypoxia time-dependent transcriptional up-regulation of SLC2A1 (that encodes the Glucose Transporter 1, GLUT-1) in all patient derived GB cells; whereas SLC2A1 expression was 10–20 fold higher in hypoxic U87 cells compared to the normoxic U87 control cells. SLC16A3 gene which encodes the monocarboxylate transporter 4 (MCT-4), was up-regulated by 5–10 fold in hypoxic UP-029 and SEBTA-003 cells, 5–15 fold in SEBTA-023 cells, and 2 fold in UP-007 and U87 cells compared to their respective normoxic controls. Moreover, CA9 transcription was highly up-regulated during hypoxia in a time-dependent manner in all GB cells investigated, approximately 10–20 fold in UP-007, SEBTA-003 and U87 cells, up to 35 fold in UP-029 and up to 70 fold in SEBTA-023 cells. Overall, these results are consistent with the Hypoxia Signaling Pathway RT2 Profiler PCR Array results (Figure 1, Figure 3 and Tables S4 and S5). Together these data reveal that hypoxia induced a potent glycolytic transcriptional response in all GB models investigated.

We noted that under normoxic conditions, the expression levels of the glycolysis related genes PDK1, PFKFB3, PFKFB4, SLC16A3, HK2, and CA9 were significantly lower relative to the housekeeping gene RPLP0, 0.02–0.04 (−25 to −50 fold), (Figure S1F–J). SLC2A1 relative expression was 0.1–0.15 (−7 to −10 fold) compared to RPLP0 in UP-007, UP-029, SEBTA-003, and SEBTA-023 cells; and only 0.025 (−40 fold) in U87 cells. Of note, we observed higher up-regulation of SLC2A1 in hypoxic U87 cells (which showed the lowest endogenous levels) compared to all other GB cells (Figure 3). Relative expression of LDHA was high in all GB cells studied, with values ranging between 0.8 to 1.2 fold compared to RPLP0 in UP-007, UP-029, SEBTA-023 and U87 cells; and a 2 fold higher expression compared to RPLP0 in SEBTA-003 cells (Figure S1F–J).

#### 3.1.3. Expression Analysis of Angiogenic Genes

**Figure 4:**
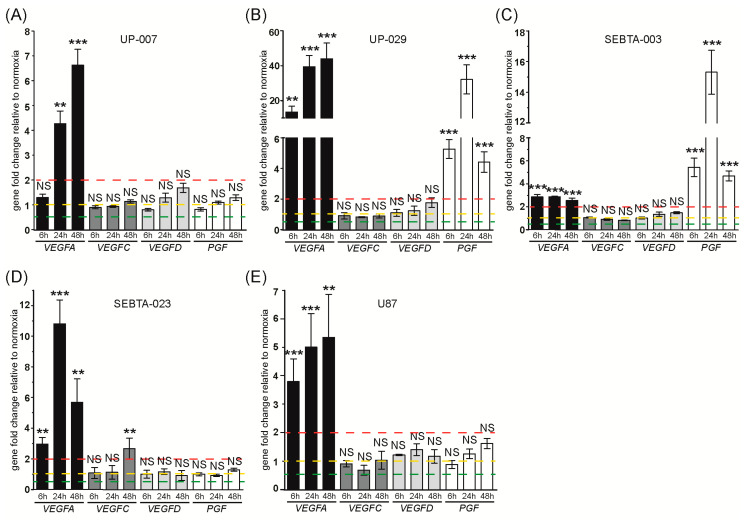
Analysis of angiogenic genes in hypoxic GB cells. (**A**) UP-007; (**B**) UP-029; (**C**) SEBTA-003; (**D**) SEBTA-023, or (**E**) U87 cells were incubated under normoxia (21% O_2_) or hypoxia (1% O_2_) for the times indicated. RNA extraction was performed and gene expression was determined by RT-qPCR. Gene expression levels were normalized to RPLP0 mRNA using the 2^-ΔΔCT^ method [13]. Error bars represent the standard deviations obtained from the median value of at least three independent experiments, each performed in triplicate. Statistical analysis was evaluated using two-tailed Student’s t-test, comparing each hypoxia time-point to the respective normoxic control. In every case a *p* value of less than 0.01 (**) and 0.001 (***) was considered statistically significant.

Angiogenic genes expression analysis confirmed the hypoxia-dependent up-regulation of VEGFA in all GB cells examined; VEGFA was induced by 15–40 fold in UP-029 cells, 2.5 fold in SEBTA-003 cells and 5–10 fold in the remaining GB cells (Figure 4A–E and Table S5). In contrast, we did not observe significant changes in VEGFC or VEGFD transcription in hypoxic GB cells, with the exception of a 2.5 fold induction of VEGFC by 48h of hypoxia in SEBTA-023 cells (Figure 4A–E and Table S5). PGF expression was unchanged in UP-007, SEBTA-023, and U87 cells, up-regulated by 5–15 fold in SEBTA-003 cells and by 5–30 fold in UP-029 cells, with the highest induction observed at 24 h post-hypoxia incubation in these cell lines (Figure 4A–E and Table S5).

The relative expression of angiogenic genes was significantly lower, between 0.08–0.01 (−12.5 to −100 fold) compared to RPLP0 in all GB cells examined (Figure S2A–E).

#### 3.1.4. Expression Analysis of Invasion Genes

**Figure 5:**
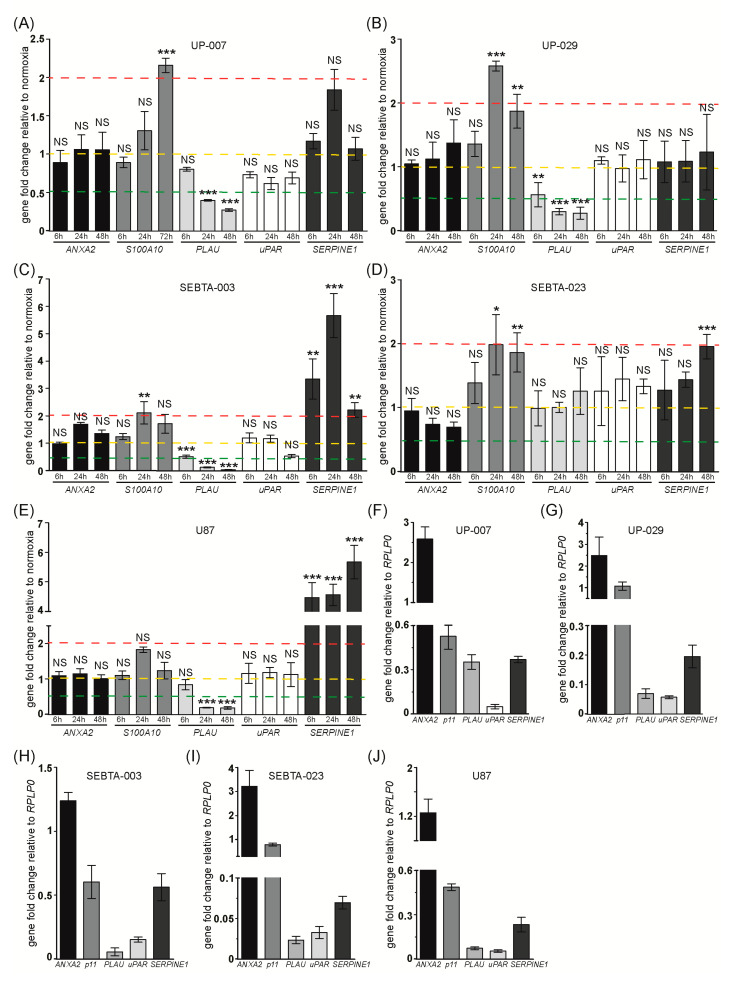
Analysis of invasion related genes in hypoxic GB cells. (**A**) UP-007; (**B**) UP-029; (**C**) SEBTA-003; (**D**) SEBTA-023; or (**E**) U87 cells were incubated under normoxia (21% O_2_) or hypoxia (1% O_2_) for the times indicated. RNA extraction was performed and gene expression was determined by RT-qPCR. Gene expression levels were normalized to RPLP0 mRNA using the 2^−ΔΔCT^ method [13]. Error bars represent the Standard Deviations obtained from the median value of at least three independent experiments, each performed in triplicate. Statistical analysis was evaluated using two-tailed Student’s *t*-test, comparing each hypoxia time-point to the respective normoxic control. In every case a *p* value of less than 0.05 (*), less than 0.01 (**) and 0.001 (***) was considered statistically significant. Relative gene expression compared to the housekeeping gene, RPLP0, under normoxic conditions for (**F**) UP-007; (**G**) UP-029; (**H**) SEBTA-003; (**I**) SEBTA-023; or (**J**) U87 cells. Relative expression was calculated by applying the following formula 2^−ΔΔCt^, where ΔCt is obtained by subtracting RPLP0 Ct value to the Ct value of our genes of interest, as indicated. Error bars represent the Standard Deviations obtained from at least three independent experiments.

We next investigated the expression of invasion genes. In agreement with our RT^2^ data, ANXA2 levels did not change during hypoxia in all GB cells investigated (Figure 5A–E and Tables S4 and S5). In contrast, expression of S100A10 that encodes the ANXA2 binding partner, was 2 fold up-regulated during hypoxia in all GB cells investigated with the exception of U87 cells. In these cells S100A10 transcription did not significantly change throughout the course of hypoxia (Figure 5A–E, Figure S3 and Table S5). Our RT^2^ PCR array data showed a significant down-regulation of PLAU that encodes the uPA protein, in UP-029 cells, relative expression of 0.4 (−3 fold) at 6 h and 0.0759 (−13 fold) by 48 h of hypoxia incubation compared to normoxic cells (Table S4). Since uPA plays a main role in tumor ECM degradation [8] we further investigated PLAU expression by RT-qPCR. We observed that hypoxia induced the down-regulation of PLAU in a time-dependent manner by 0.5 to 0.1 (−2 to −10 fold) in UP-007, UP-029, SEBTA-003 and U87 cells (Figure 5A–E). Consistent with our RT^2^ PCR array data, no changes in PLAU transcription were observed in hypoxic SEBTA-023 cells (Figure 5A–E and Table S4). We next analyzed the expression of the uPA receptor, uPAR. We did not observe significant changes in uPAR transcription during hypoxia in any of the GB cells investigated (Figure 5A–E). Broadening these studies, analysis of SERPINE1 which encodes the PAI-1 protein, showed a 2 fold up-regulation of this gene at 48 h post-hypoxia incubation in SEBTA-023 cells and a 3–6 fold induction in hypoxic SEBTA-003 and U87 cells. SERPINE1 expression did not change in UP-007 or UP-029 cells during the hypoxia time-course compared to their matched normoxic control cells (Figure 5A–E). MMPs transcriptional analysis showed that MMP-2 expression did not significantly change during hypoxia in UP-007, SEBTA-023 or U87 cells (Figure S4A,D,E). We observed a 2–3 fold induction of MMP-2 in hypoxic UP-029 cells, whereas we were not able to detect MMP-2 transcript in SEBTA-003 cells under our experimental conditions (Figure S4B-C). MMP-9 expression did not significantly change during hypoxia in UP-007, UP-029, and SEBTA-003 cells (Figure S4A–C). Surprisingly, we observed a 0.4–0.5 (−2 to −3 fold) hypoxia-dependent down-regulation of MMP-9 in SEBTA-023 and U87 cells (Figure S4D–E).

Relative expression analysis of the invasion genes compared to RPLP0 showed that both ANXA2 and S100A10 mRNAs are abundant in all GB cells studied (Figure 5F–J). ANXA2 relative expression compared to RPLP0 was 2.5 fold higher in UP-007 and UP-029 cells, 1.25 fold higher in SEBTA-003 and U87 cells, and 3 fold higher in SEBTA-023 cells (Figure 5F–J). Of all genes investigated, ANXA2 showed the highest relative expression compared to RPLP0 in all GB cells analyzed; with the exception of LDHA expression in SEBTA-003 cells (Figure S1). Together these data indicate that ANXA2 mRNA is highly abundant in GB cells. Based on these findings, we next analyzed ANXA2 protein expression in our GB cells by Western blotting (Figure S5). We also included the non-neoplastic astrocyte cell line, SC-1800, and the breast cancer cell line, MDA-MB-231 that we had previously shown to express very high levels of ANXA2 [14]. These results revealed that ANXA2 expression was similar or higher in SC-1800 and GB cell lines compared to MDA-MB-231 cells (Figure S5). S100A10 relative expression compared to RPLP0 was 1.1 fold higher in UP-029, 0.8 (−1.25 fold) in SEBTA-023 cells, and 0.5–0.6 (−1.7 to −2 fold) in UP-007, SEBTA-003 and U87 cells (Figure 5F–J). Analysis of S100A10 protein expression demonstrated that this protein was more highly expressed in SC-1800 non-neoplastic astrocytes and in UP-007, UP-029 and SEBTA-023 cells compared to MDA-MB-231 cells; while SEBTA-003 cells expressed lower levels of S100A10 and U87 showed similar S100A10 protein expression compared to MDA-MB-231 cells (Figure S5). SERPINE1 expression was 0.25–0.5 (−2 to −4 fold) compared to RPLP0 in all GB cells examined, except in SEBTA-023 cells where SERPINE-1 expression was only 0.07 (−15 fold) compared to RPLP0 (Figure 5F–J). PLAU expression was 0.35 (−2.8 fold) compared to RPLP0 in UP-007 cells, and 0.02–0.07 (−15 to −50 fold) compared to RPLP0 in the remaining GB cells (Figure 5F–J). uPAR transcription was highest in SEBTA-003 cells, 0.17 (−6 fold) compared to RPLP0 and between 0.03-0.06 (−17 to −33 fold) compared to RPLP0 in the remaining GB cells (Figure 5F–J).

#### 3.1.5. Expression Analysis of Other Hypoxia Related Genes

**Figure 6:**
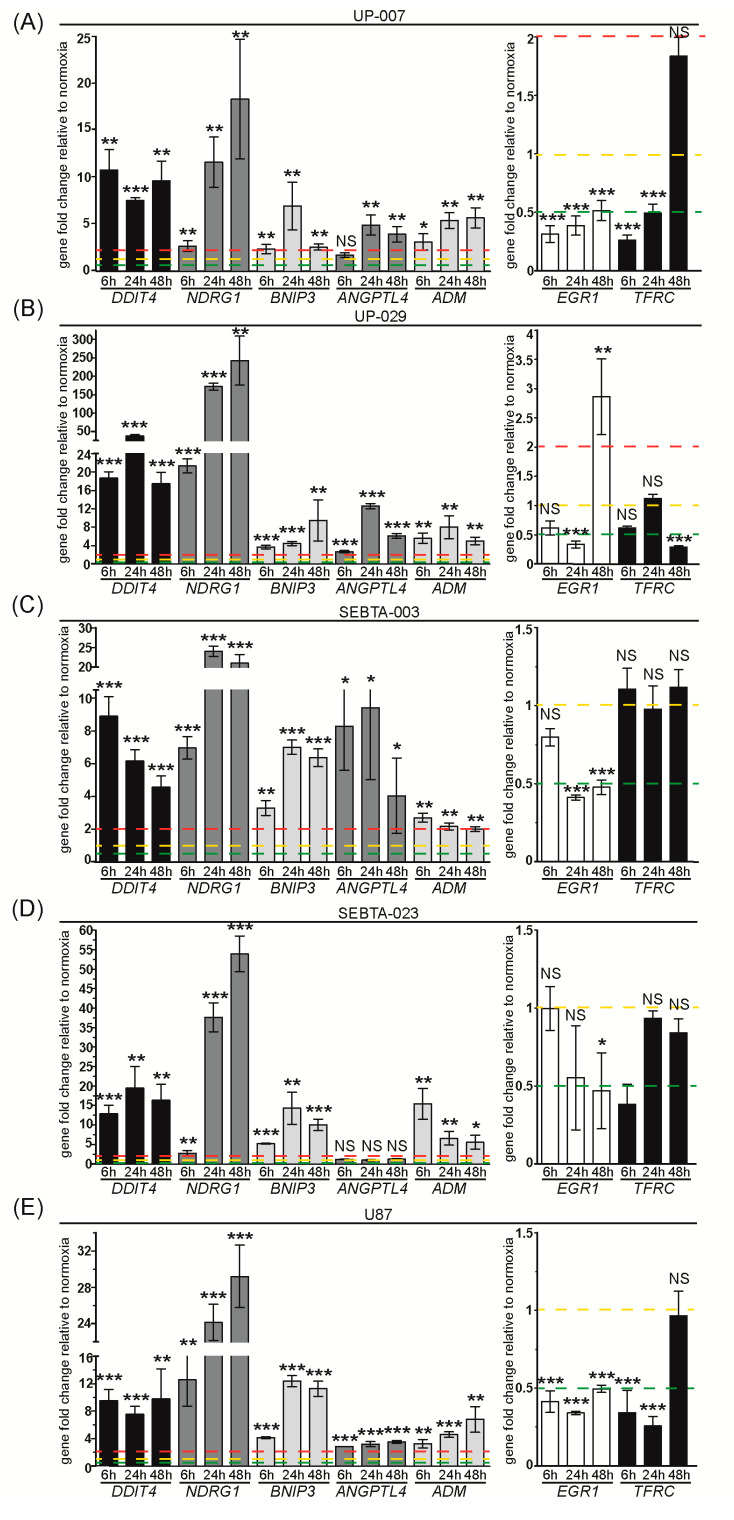
Analysis of varied functions genes in hypoxic GB cells. (**A**) UP-007; (**B**) UP-029; (**C**) SEBTA-003; (**D**) SEBTA-023; or (**E**) U87 cells were incubated under normoxia (21% O_2_) or hypoxia (1% O_2_) for the times indicated. RNA extraction was performed and gene expression was determined by RT-qPCR. Gene expression levels were normalized to RPLP0 mRNA using the 2^−ΔΔCT^ method [13]. Error bars represent the Standard Deviations from the median value of at least three independent experiments, performed in triplicate. Statistical analysis was evaluated using two-tailed Student’s t-test, comparing each hypoxia time-point to the respective normoxic control. In every case a *p* value of less than 0.05 (*), less than 0.01 (**) and 0.001 (***) was considered statistically significant.

We analyzed the transcription of genes that encode proteins with diverse cellular functions outside glycolysis, angiogenesis, and invasion cellular processes, and that were shown to be differentially expressed in hypoxic UP-029 and SEBTA-023 cells using our RT2 Profiler PCR Array analysis (Figure 1F and Table S4). These results are depicted in Figure 6 and Table S5.

We observed a significant 5–10 fold hypoxia-dependent up-regulation of DDIT4 (that encodes the protein Regulated in Development and DNA Damage responses 1, REDD1) in UP-007, SEBTA-003 and U87 cells; 10–20 fold induction in SEBTA-023 cells and 15–40 fold induction in UP-029 cells compared to their respective normoxic control cells. NDRG1 was highly induced during hypoxia in a time-dependent manner in all GB cells analyzed, with expression levels ranging from 3–18 fold in UP-007, 20–240 fold in UP-029, 7–25 fold in SEBTA-003, 3-55 fold in SEBTA-023 and 10–30 fold in U87 cells compared to their normoxic control cells. BNIP3 expression was 3–10 fold up-regulated during hypoxia in UP-007, UP-029 and SEBTA-003 cells, and 5–15 fold in SEBTA-023 and U87 cells. ANGPTL4 transcription was 2-5 fold higher during hypoxia in UP-007 and U87 cells and 3–10 fold higher in hypoxic UP-029 and SEBTA-003 cells. We did not observe any significant changes in ANGPTL4 mRNA levels in SEBTA-023 cells throughout the course of hypoxia. ADM expression was 3-8 fold higher during hypoxia in UP-007, UP-029 and U87 cells, 5–15 fold higher in hypoxic SEBTA-023 cells and 2 fold up-regulated in hypoxic SEBTA-003 cells compared to the respective normoxic controls. We observed the down-regulation of EGR1 during hypoxia by 0.4–0.5 (−2 to −2.5 fold) in all GB cells analyzed. Interestingly, in UP-029 cells after an initial hypoxic down-regulation of EGR1 transcription, we observed a 3 fold induction of EGR1 by 48 h of hypoxia; this was also detected in the RT2 Profiler PCR array (Figure 1F). We observed the down-regulation of TFRC by 0.3–0.5 (−2 to −3 fold) in UP-007, SEBTA-023 and U87 cells up to 24 h of hypoxia, while TFRC expression was similar to their normoxic control cells by 48 h of hypoxia in these cells. In UP-029 cells we observed the down-regulation of TFRC by 0.25 (−4 fold) at 48 h of hypoxia compared to normoxic control cells. TFRC expression did not significantly change in SEBTA-003 cells during the course of hypoxia. Overall, the data presented above was consistent with the results obtained in the Hypoxia Signaling Pathway RT2 Profiler PCR Array (Figure 1F and Table S4).

Relative gene expression analysis showed that BNIP3 and TFRC genes were expressed at 0.1–0.2 (−5 to −10 fold) compared to RPLP0 in all GB cells studied. NDRG1 relative expression was 0.1 (−10 fold) in UP-007, 0.015 (−65 fold) in UP-029, 0.2 (−5 fold) in SEBTA-003, 0.005 (−200 fold) in SEBTA-023 and 0.04 (−25 fold) in U87 cells compared to RPLP0. While DDIT4, EGR1, and ADM were expressed at levels lower than 0.04 fold (−25 fold) as compared to RPLP0 in all GB cells. ANGPTL4 expression was 0.2 (−5 fold) in SEBTA-003, 0.04-0.06 (−17 to −25 fold) in UP-007, UP-029 and U87 cells and 0.01 (−100 fold) in SEBTA-023 cells compared to RPLP0. In summary, we observed low to very low expression of these genes under normoxic conditions that were substantially impacted following GB exposure to hypoxic conditions.

### 3.2. Expression of Hypoxia Related Proteins in GB Cells

Having noted a potent, conserved transcriptional response to hypoxia in our various GB cell models, we next questioned if this data was conserved at the protein level. We were also interested in investigating the possibility of hypoxia induced protein regulation in cases where gene transcription remained unchanged throughout the course of hypoxia.

**Figure 7:**
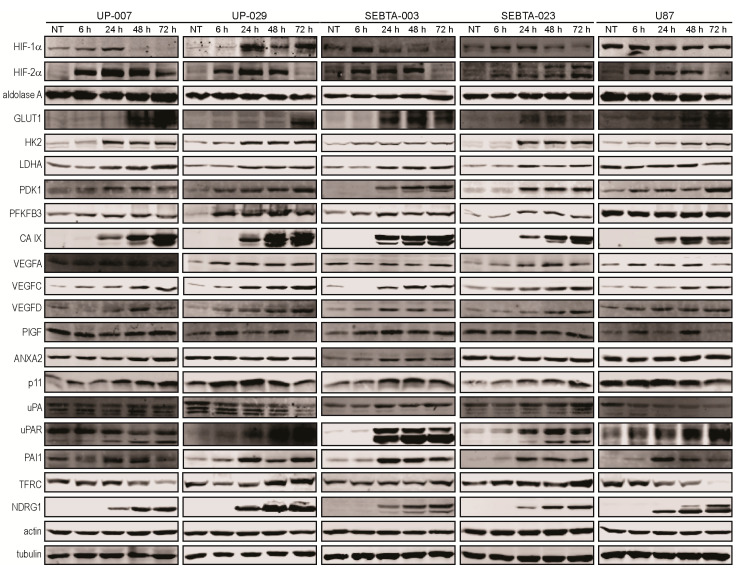
Analysis of hypoxia related proteins in GB cells. UP-007; UP-029; SEBTA-003; SEBTA-023 or U87 cells were incubated under normoxia (21% O_2_) or hypoxia (1% O_2_) for the times indicated. Cells were lysed and 20 µg of each protein extract was subjected to SDS-PAGE, and analyzed by Western blotting with the antibodies indicated. NT- non treated/ normoxia.

Our studies revealed the accumulation of both HIF-1α and HIF-2α proteins during hypoxia in all GB cell lines analyzed, except U87 cells. In this cell line, HIF-1α protein expression remained unchanged throughout our hypoxia experimental time-course (Figure 1A,B, Figure 2A–C and Figure 7).

We observed hypoxia dependent up-regulation of all glycolytic proteins analyzed, namely GLUT1, HK2, LDHA, PDK1, PFKFB3, and CA IX in our GB cell lines; with the exception of LDHA and PFKFB3 proteins in the commercially available cell line, U87 (Figure 7). We also included aldolase A protein in our Western blot analysis. ALDOA transcription did not change during hypoxia (Table S4). In agreement with the transcriptional data, we did not observe any significant changes in aldolase A protein levels during hypoxia in all GB cell lines analyzed (Figure 7). Overall, the Western blotting data was in agreement with our gene expression analysis, meaning that up-regulation of the glycolytic proteins was at least in part due to hypoxic transcriptional regulation of the genes that encode these proteins.

Western blot analysis of angiogenic proteins showed hypoxia-dependent up-regulation of VEGFA in UP-007, UP-029, SEBTA-023, and U87 cells (Figure 7). We did not observe changes in VEGFA expression in SEBTA-003 cells during hypoxia (Figure 7). Interestingly, we observed the up-regulation of both VEGFC and VEGFD protein levels during hypoxia in all GB cell lines investigated, even though the transcription of these genes was unchanged (Figure 4 and Figure 7). Finally, we observed a hypoxic up-regulation of PlGF expression in UP-029 and SEBTA-003 cells, and no significant change in PlGF levels in UP-007, SEBTA-023 and U87 cells during the hypoxia time-course (Figure 7). These results are consistent with the RT-qPCR data, indicating that transcriptional up-regulation of PGF during hypoxia in UP-029 and SEBTA-003 cells leads to increased PlGF protein levels in these cells.

We examined the expression of invasion proteins by Western blotting. These results showed that ANXA2 levels did not change during hypoxia in UP-007, UP-029, SEBTA-023 and U87 cells, consistent with the RT-qPCR results (Figure 5A–E and Figure 7). We observed up-regulation of ANXA2 in SEBTA-003 cells during hypoxia compared to normoxic control cells (Figure 7). RT-qPCR analysis showed a 1.8 fold up-regulation of ANXA2 at 24 h of hypoxia in SEBTA-003 cells which could at least in part explain ANXA2 increased protein expression during hypoxia in these cells (Figure 5C). S100A10 protein expression increased in UP-007, UP-029, SEBTA-003, and SEBTA-023 cells, but not in U87 cells during hypoxia (Figure 7). These results are in accordance with the RT-qPCR data, indicating that S100A10 is transcriptionally regulated during hypoxia leading to increased S100A10 protein levels. uPA levels did not change in UP-007, SEBTA-003, and SEBTA-023 cells during hypoxia. In contrast, uPA was down-regulated in hypoxic UP-029 and U87 cells (Figure 7). Interestingly, we observed up-regulation of uPAR during hypoxia in all GB cells analyzed with the exception of UP-007 cells (Figure 7). These data contrast the RT-qPCR data that showed no changes in uPAR transcription during hypoxia (Figure 5A–E). Together, these results indicate that uPAR is being post-transcriptionally regulated in UP-029, SEBTA-003, SEBTA-023, and U87 cells during hypoxia. Similarly to uPAR, PAI-1 was up-regulated during hypoxia in all GB cells with the exception of UP-007 cells (Figure 7). These results are in agreement with unchanged SERPINE1 expression in hypoxic UP-007 cells and increased SERPINE1 transcription in SEBTA-003, SEBTA-023, and U87 cells during hypoxia. We did not observe any changes in SERPINE1 transcription in UP-029 cells during hypoxia even though there was a clear up-regulation at the protein level. These results suggest different mechanism(s) for SERPINE-1/PAI-1 regulation during hypoxia in the various GB cells studied.

Analysis of NDRG1 expression by Western blotting showed a significant time-dependent up-regulation of this protein during hypoxia in all GB cells analyzed. This was consistent with our RT-qPCR data (Figure 6 and Figure 7). We observed a time-dependent down-regulation of TFRC in UP-007 and U87 cells during hypoxia, a transient down-regulation of TFRC at 6 and 24 h post-hypoxia in UP-029 cells and no change in TFRC expression in SEBTA-003 and SEBTA-023 cells during hypoxia compared to normoxic conditions (Figure 7). Overall, these results were in agreement with our RT-qPCR data, indicating that TFRC is transcriptionally regulated during hypoxia (Figure 6).

### 3.3. Correlation of Hypoxia Altered Genes with Clinical Outcomes in GB Patients

**Figure 8:**
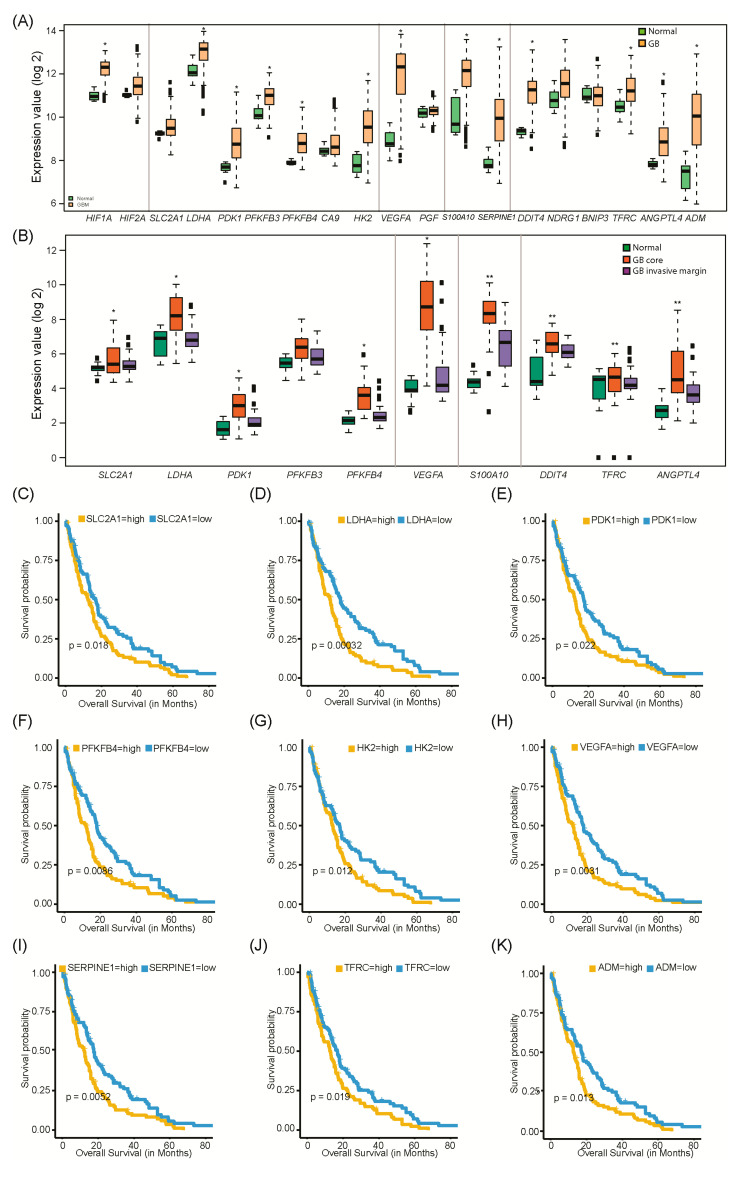
Expression of hypoxia-related genes in GB patients. (**A**) Expression of selected hypoxia-related genes in normal brain and GB patient samples. Data sourced from the published study with gene expression microarrays generated from 7 normal brain and 217 GB tumors. Normalized expression values on log_2_ scale are indicated on the *y*-axis. In the boxplots, the top, middle and bottom box delimiters represent the 75th, 50th, and 25th percentiles of the data, respectively. Top and bottom whiskers show the 75th percentile + 1.5*interquartile range and 25th percentile – 1.5*interquartile range, respectively. The ‘*’ above the boxplot indicates that the change in gene expression between normal and GB samples are significant at false discover rate, FDR < 0.05. (**B**) Expression of subset of hypoxia-related genes in non-neoplastic brain tissue samples (*n* = 17), contrast-enhancing GB core samples (*n* = 39) and non-enhancing GB invasive margin samples (*n* = 36). Normalized expression values on log2 scale are indicated on the *y*-axis and were obtained from the RNA-Seq data of the published study. The ‘*’ above the boxplot indicates that the change in gene expression between normal and GB core samples are significant at false discover rate, FDR < 0.05, whereas the “**” denotes the significance between normal and GB core as well as normal and GB invasive margin samples. (**C**–**K**) Kaplan–Meier survival curves of GB patients (*n* = 217) divided in high and low expression levels for each gene (split by median expression) as indicated. Survival curves were compared using the log-rank test. Genes that show significant association (*p* < 0.05) with the overall survival are shown.

To determine the clinical relevance of the genes that were differentially expressed in our GB cell lines during hypoxia, we examined their expression patterns in GB patient datasets. For this analysis, we primarily used the molecular analysis of brain neoplasia dataset (E-MTAB-3073). This is the largest microarray dataset and includes normal controls. In agreement with the data obtained with our GB in vitro lines, nearly all tested genes (17 out of 18) showed differential expression in GB patient samples compared to normal brain tissue (Figure 8A).

We observed that HIF-1α and HIF-2α expression were up-regulated in GB patient samples as compared to normal tissue which is in agreement with the presence of hypoxic cores in GB tumors (Figure 8A). We were able to detect HIF-1α and HIF-2α proteins even under normoxic conditions in our GB cells (Figure 1, Figure 2 and Figure 7). Moreover, the relative expression of both genes (as compared to RPLP0) was high (Figure S1).

In agreement with our RT-qPCR data (Figure 3), the database analysis of the gene expression study showed up-regulation of the glycolytic genes: SLC2A1, LDHA, PDK1, PFKFB3, PFKFB4, HK2, and CA9 in GB samples as compared to non-neoplastic brain tissue (Figure 8A). Importantly, expression levels of SLC2A1, LDHA, PDK1, PFKFB3, and PFKFB4 were high in GB hypoxic core when we compared the gene expression profiling of GB core, GB invasive margin and normal tissue (Figure 8B).

We observed increased expression of the angiogenic genes VEGFA and PGF in GB patient samples as compared to non-neoplastic brain tissue (Figure 8A). VEGFA expression was significantly higher in GB hypoxic core regions but not in the GB invasive margin compared to normal tissue (Figure 8B). The transcription of S100A10 and SERPINE1 both associated with tumor invasion, was up-regulated in GB patients compared to non-neoplastic control samples (Figure 8A). Interestingly, S100A10 expression was higher in both GB core and invasive margin regions compared to normal brain tissue (Figure 8B). These data suggest an important role for S100A10 in GB invasiveness which might be triggered by hypoxia. To support this hypothesis, we observed higher expression of S100A10 in GB hypoxic core samples compared to the GB invasive margin (Figure 8B). Finally, we observed significant over expression of DDIT4, NDRG1, TFRC, ANGPTL4, ADM, and BNIP3 in GB patient samples compared to non-neoplastic brain tissue (Figure 8A). Both DDIT4 and ANGPTL4 expression were higher in GB core and invasive margin versus normal brain tissue, and more elevated in GB hypoxic core regions as compared to the GB invasive margin (Figure 8B). Overall, these results were in agreement with our in vitro transcription data; with the exception of TFRC that was either down-regulated or unchanged during hypoxia in the GB cells (Figure 6).

Gene expression analysis in Low Grade Gliomas (LGG) compared to GB or normal brain tissue showed that the majority of the glycolysis related genes, namely LDHA, PDK1, SLC2A1, and CA9, were expressed at similar or even slightly lower levels in LGG compared to normal brain tissue; whereas PFKFB4 expression increased in a tumor grade dependent manner and PFKFB3 expression was similar in LGG and GB (Figure S6). ADM, ANGPTL4, TFRC, NDRG1, and BNIP3 expression were also similar in LGG compared to normal brain tissue (Figure S6). On the other hand, expression of DDIT4 and PGF were similar in LGG and GB (Figure S6). Importantly, expression of the invasion and angiogenesis genes, S100A10, SERPINE1, and VEGFA, increased with tumor grade, and this was also observed for HIF-1α (Figure S6). Together these results are consistent with increasing hypoxia and aggressive phenotype in GB compared to LGG.

We next analyzed if differential expression of the aforementioned genes could be linked to clinical outcome, specifically with patient overall survival. We observed that GB patients showing high expression of SLC2A1, LDHA, PDK1, PFKFB4, HK2, VEGFA, SERPINE1, and ADM genes had significantly worse overall survival (Figure 8C–K).

## 4. Discussion

GB is the deadliest type of primary malignant brain tumor. A key characteristic of these tumors is the presence of hypoxic cores associated with GB aggressiveness and chemo-resistance. Solid tumors are continually subjected to different types of hypoxic stresses including acute, chronic and cycling hypoxia (also known as intermittent hypoxia). Acute hypoxia typically happens when small blood vessels shut down often due to restrictions caused by the increased tumor mass or the irregular flow of red blood cells. Acute hypoxia usually lasts for a few minutes to a few hours and can be easily reversed. On the other hand, chronic hypoxia lasts for prolonged periods of time (>24 h) and may result in cell death. Chronic hypoxia occurs as a result of over-proliferation of cancer cells leading to enhanced cellular density and increasing distance between a proportion of the tumor cells and the nearest blood vessels. Cyclic hypoxia is a consequence of the transient shut down of the disorganized and easily collapsible tumor vasculature resulting in intermittent hypoxia which can last from periods of minutes to several days [15]. Of note, cyclic hypoxia has been shown to enhance several hallmarks of cancer compared to chronic hypoxia, including angiogenesis, immune evasion, metastasis and survival [15]. This indicates that the constant cycling between normoxic and hypoxic conditions which is frequently observed during tumor growth plays a main role in cancer progression.

Here, we characterized the response of several patient-derived GB cell lines to hypoxia, at the protein and transcript level, to better understand how GB cells adapt to this important microenvironmental stress. We investigated the expression of 92 hypoxia related genes in our in vitro GB cell models, and examined these results alongside publicly available clinical datasets, to identify potential molecular markers and/or therapeutic targets. These can be analyzed in the future for the development of novel and more effective therapies for GB patients.

RT-qPCR analysis of the main transcription factors involved in the regulation of the hypoxic response showed the down-regulation of *HIF-1α* in hypoxic UP-007, UP-029, and SEBTA-003 cells while no significant change was observed in either SEBTA-023 or U87 cells during hypoxia. In contrast, *HIF-2α* transcription was up-regulated in a time-dependent manner during hypoxia in UP-007, UP-029 and SEBTA-023 cells, and remained unchanged in SEBTA-003 and U87 cells (Figure 2D–H). Our protein expression data showed the induction of both HIF-1α and HIF-2α during hypoxia in all primary biopsy derived GB cells (Figure 1A,B, Figure 2A,B and Figure 7). Noticeably, in the widely studied U87 cells, HIF-1α protein expression did not change, whereas HIF-2α was up-regulated during hypoxia (Figure 2C and Figure 7); this could potentially provide some information regarding which genes are regulated by HIF-1α and/or HIF-2α in GB cells. However, we have to consider that other regulatory mechanisms are likely triggered by hypoxia that can enhance HIF-1 transcriptional activity even if the total level of the HIF-1α protein is unchanged. For example, under normoxia the Factor Inhibiting HIF (FIH) hydroxylates Asp 803 within the HIF-α subunit preventing the interaction between HIF and coactivators; while during hypoxia, FIH is inhibited and this post-translational modification of HIF-1α does not occur [5,6]. HIF-1 transcriptional activity also increases during hypoxia due to decreased cellular NAD+ which downregulates Sirt1 leading to enhanced HIF-1α acetylation [16].

We were able to detect HIF-1α and HIF-2α proteins in the GB cells under normoxic conditions (Figure 1A–C and Figure 2A–C, and Figure 7). We hypothesized that this occurred due to the enhanced activation of the PI3K/AKT signaling pathway which is typically observed in GB [5]. *EGFR* gene amplification and/or overexpression are frequently observed in GB. The most common *EGFR* gene mutation being the deletion of exons 2–7, resulting in a truncated and constitutively active, ligand independent receptor, EGFRvIII [5]. EGFR stimulation either by ligand binding and/or gene amplification results in the activation of the PI3K pathway, leading to increased HIF-1α translation and stabilization via the PI3K/AKT/FRAP/mTOR pathway [17,18,19]. Western blot analysis showed that the PI3K/AKT signaling pathway (assessed by AKT activation, P-AKT immuno-blot) was highly up-regulated in all GB cells studied in comparison with the SC-1800, non-neoplastic astrocyte cell line, and even when compared to the breast carcinoma cell line, MDA-MB-231 (Figure S5). Furthermore, we did not observe any changes in the activation of the PI3K/AKT or the MAPK/ERK 1/2 signaling pathways, assessed by P-AKT and P-ERK 1/2 Western blotting, respectively, throughout our hypoxia time-course (Figure S7).

The majority of the literature highlights HIF-1α as the main hypoxia transcriptional regulator in GB. Our findings, demonstrate the transcriptional up-regulation of *HIF-2α* during hypoxia in UP-007, UP-029, and SEBTA-023 cells, and HIF-2α protein accumulation in all GB cell lines investigated. These data provide novel and important information that suggest a potentially important role for HIF-2α in regulating the hypoxia response in GB. Moreover, our results indicate that clinical approaches that are focused solely in HIF-1α inhibition (e.g., EZN-2968, CAY10585, NSC 607097) might not constitute robust therapies against GB, particularly as HIF-2α expression will not be affected and could, independent of HIF-1α, orchestrate the GB hypoxic response.

Our in vitro studies showed a significant hypoxia-dependent up-regulation of 8 glycolysis related genes/proteins in all GB cell lines investigated. These include enzymes that are responsible for the initial steps of glycolysis, namely HK2 that catalyzes the first step of glycolysis, conversion of glucose to glucose-6-phosphate; PFKFB3 and PFKFB4 that catalyze the conversion of fructose-6-phosphate to fructose-1,6-biphosphate. We further observed the up-regulation of LDHA that catalyzes the conversion of the final product of glycolysis, pyruvate to lactate; and the enzyme PDK1 which inactivates Pyruvate Dehydrogenase (PDH), inhibiting the conversion of lactate to acetyl-coA, which is required for the TCA cycle and oxidative phosphorylation (Figure 3 and Figure 7). We also observed hypoxic up-regulation of the glucose transporter, *SLC2A1*/GLUT-1 in all GB cell lines investigated (Figure 3 and Figure 7), confirming previous studies [20]. Low oxygen availability forces the cells to rely on glycolysis for energy and metabolic intermediates production. Glycolysis produces 18 fold less ATP compared to mitochondrial respiration. Consequently, an increase in the intracellular levels of glucose during hypoxia is essential for survival of the GB cells. Glycolysis intermediates can be quickly diverted to anabolic pathways as substrates for DNA replication, lipid and protein biosynthesis, which are required by the rapid proliferating GB cells. We observed the up-regulation of *SLC16A13* and *CA9* that encode for MCT-4 and CA IX proteins, respectively. These proteins are involved in intracellular pH regulation. MCT-4 is responsible for the export of the lactate formed during glycolysis as well as the efflux of protons; while CA IX catalyzes the formation of bicarbonates and protons, both leading to extracellular acidification [21]. In this way, both MCT-4 and CA IX also promote ECM degradation due to acidosis of the tumor microenvironment, contributing to GB invasion. We did not observe significant change in the mRNA levels of the following glycolytic related genes as assessed by the Hypoxia Signaling RT2 array in hypoxic SEBTA-023 and UP-029 cells compared to their respective normoxic controls: *ALDOA*, *ENO1*, *GPI*, *PFKL*, *PFKP*, *PGK1*, *PGAM1*, *PKM*, *SLC2A3/GLUT-3*, or *TPI-1* (Table S4). Analysis of GB patient samples showed up-regulation of all glycolytic genes identified in our in vitro experiments with the exception of *SLC16A3* (Figure 8A). Importantly, while these genes were over-overexpressed in GB hypoxic core region, their expression in the GB invasive margin was similar to normal brain tissue (Figure 8B). These results suggest a dynamic hypoxia-dependent regulation of the glycolysis related genes in GB which may play an important role during periods of cycling (intermittent) hypoxia. Further studies are necessary to investigate this hypothesis. Furthermore, in vivo clinical data analysis also showed that high expression of *SLC2A1*, *LDHA*, *PDK1*, *PFKFB4*, and *HK2* mRNAs in GB were all associated with significantly worse patient overall survival (Figure 8C–G).

A previous study showed the up-regulation of *PFKFP*, *PDK1*, *PGAM1*, *ENO1*, *HK2*, *ALDOA*, and *ENO2* during hypoxia in 5 patient derived biopsy cell lines and two commercially available cell lines (U87 and U251) by RT-qPCR [22]. The authors further demonstrated that these genes were important for GB growth in vitro and in vivo using a NOD/SCID mouse model [22]. In contrast, we did not observe significant changes in *PFKFP*, *PGAM1*, *ENO1*, or *ALDOA* in hypoxic GB cells.

Taken together, these results suggest that glycolytic proteins can potentially constitute promising molecular markers and/or therapeutic targets for GB treatment. The CA IX inhibitor, Indisulam, is currently being evaluated in clinical trials for the treatment of a range of cancers, including metastatic melanoma, lung, acute myeloid leukemia (AML) and metastatic breast cancer where brain metastasis is commonly observed [5].

Analysis of angiogenic genes and proteins confirmed the up-regulation of VEGFA in hypoxic GB cells (Figure 4 and Figure 7) [23,24,25]. We observed *PGF* mRNA and PlGF protein up-regulation in hypoxic UP-029 and SEBTA-003 cells (Figure 4 and Figure 7), but not in the remaining patient derived GB cells investigated compared to their respective normoxic control cells. Interestingly, we did observe up-regulation of PlGF protein levels in U87 cells (Figure 7). Taken together, these results suggest that the hypoxic regulation of *PGF/*PlGF in GB is not a general mechanism, and could occur at a transcriptional and/or protein level in different GB cells. Stratification of patients taking into account the hypoxic induction of *PGF/*PlGF should be considered in the future, as this could potentially have significant repercussions in clinical outcome, particularly when considering anti-angiogenic therapy. Interestingly, whereas we did not observe significant hypoxia dependent changes in *VEGFC* and *VEGFD* transcription, their protein levels were up-regulated in a hypoxia time-dependent manner in all GB cell lines investigated (Figure 4 and Figure 7). Clinical data analysis showed up-regulation of *VEGFA* and *PGF* in GB patient samples (Figure 8A). Similarly to what was observed for the glycolytic genes, *VEGFA* expression was higher in the GB hypoxic core region but not in the GB invasive margin compared to normal brain tissue (Figure 8B), indicating a dynamic transcription of *VEGFA* which is essential for triggering angiogenesis when tumors are subjected to hypoxia. We also observed that high-expression of *VEGFA* was associated with significantly worse overall survival in GB patients (Figure 8H).

Antiangiogenic treatment of GB patients with the VEGFA monoclonal antibody bevacizumab (Avastin) has been used as mainstay of salvage therapy [26]. Our in vitro data support that anti-angiogenic treatment focused solely on VEGFA, might overlook the roles of PlGF, VEGFC, and VEGFD in GB angiogenesis. This could, at least in part, suggest why anti-VEGFA therapy has fallen short from the initially anticipated efficacy. Furthermore, we have to consider that anti-VEGFA therapy might in fact exacerbate the roles of PlGF, VEGFC, and VEGFD in GB angiogenesis.

Analysis of invasion associated genes showed a significant induction of *S100A10* in all biopsy derived GB cells and of *SERPINE1* in a subset of the GB cell lines analyzed (Figure 5A–E). Tumor invasion is a major cause of GB chemoresistance and patient death. It was surprising to observe that hypoxia did not enhance the expression of the majority of the invasion genes analyzed, including *MMP-2*, *MMP-9*, *PLAU*/uPA, *CTSA* (cathepsin A), *ANXA2*, or *uPAR*.

A previous publication reported that HIF-1α siRNA knockdown led to *MMP-2* downregulation in U87, U251, U373 and LN18 GB cells [27]. Another study showed that HIF induces TGF-β2, leading to the upregulation of *MMP-2* and *MMP-9* expression in human glioma cells [28]. In contrast, we did not observe significant *MMP-2* or *MMP-9* induction in most of our GB cell lines during hypoxia, with the exception of a 2–3 fold induction of *MMP-2* in hypoxic UP-029 cells (Figure S4B). On the contrary, we observed a 0.4–0.5 (−2 to −3 fold) hypoxia-dependent down-regulation of *MMP-9* in SEBTA-023 and U87 cells (Figure S4D,E). Endogenous expression of these genes was very low (<−500 fold) compared to *RPLP0* (data not shown) and we were not able to detect MMP-2 or MMP-9 proteins in our GB cells. Taken together, these data indicate that GB cells have low endogenous levels of *MMP-2* and *MMP-9* and that hypoxia does not induce a potent *MMP* response in GB cells.

On the other hand, *ANXA2* and *S100A10* mRNAs were highly expressed in all GB cells investigated (Figure 5F–J), translating into the high expression of these proteins in GB cells (Figure S5). S100A10 protein expression was up-regulated during hypoxia in all biopsy derived GB cells investigated; whereas the expression of ANXA2 remained unchanged in most GB cells, with the exception of SEBTA-003 cells where ANXA2 protein levels increased during hypoxia (Figure 7). The ANXA2-S100A10 heterotetramer constitutes an important plasminogen receptor that plays a major role in plasmin activation at the surface of many cancer cells [7,8]. ANXA2 is a multi-functional protein that can exist as a monomer or as the ANXA2-S100A10 heterotetramer [7,14,29,30], while S100A10 protein stability is highly dependent on its association with ANXA2, being quickly ubiquitinated and degraded via the proteasome in the absence of ANXA2 [7]. In this way, an increase in S100A10 expression will correspond to an increase in the levels of the ANXA2-S100A10 heterotetramer. Importantly, *S100A10* expression was highly up-regulated in GB clinical samples as compared to non-neoplastic brain tumor (Figure 8A), and higher expression of *S100A10* was observed in both GB hypoxic core region and invasive margin compared to normal brain tissue (Figure 8B). Together, these results suggest that S100A10 might play an important role in GB invasion. Further studies are necessary to investigate this hypothesis.

We observed the hypoxia-dependent up-regulation of *SERPINE1/*PAI-1 (gene and protein levels) in SEBTA-003, SEBTA-023, and U87 cells, while no changes were observed in hypoxic UP-007 or UP-029 cells (Figure 5 and Figure 7). Interestingly, even though we did not observe transcriptional up-regulation of *SERPINE1* in hypoxic UP-029 cells, we did observe a hypoxia-dependent increase in PAI-1 protein levels in this cell line (Figure 7). Although the over-expression of PAI-1, the main inhibitor of the plasmin activation system, might seem opposing to a more aggressive cancer cell phenotype, this protein has been shown to exhibit several pro-tumorigenic functions [31]. High levels of PAI-1 have been associated with shorter overall survival and poor prognosis in many cancers including breast, gastric, colorectal, pancreas, glioma, lung, kidney, prostate, liver, and bone [31,32,33,34]. Here, we confirmed the association of high *SERPINE1* expression with significantly worse GB patient overall survival (Figure 8I). PAI-1 has been shown to promote cell growth via different mechanisms. PAI-1 inhibition of fibrinolysis, via inactivation of tPA and uPA, leads to maintenance of thrombin activity which is able to interact with Protease-Activated Receptors (PAR) in cancer cells enhancing PAR-dependent proliferation; PAI-1 has also been shown to stimulate fibronectin-dependent cell growth [31]. PAI-1 also plays a role in cancer cell death resistance via inhibition of caspase 3, protecting tumor cells from chemotherapy-induced apoptosis. Extracellular PAI-1 inhibits the cleavage of Fas Ligand (FasL) by plasmin at the surface of cancer cells protecting them from FasL-mediated apoptosis and chemotherapy induced cell death. Furthermore, PAI-1 interacts with Low density Lipoprotein Receptor-related Protein 1 (LRP-1), inducing c-Jun/ERK signaling and the subsequent increase in the expression of anti-apoptotic proteins such as Bcl 2 and Bcl-XL [31]. PAI-1 has pro-angiogenic activity promoting migration, survival and proliferation of endothelial cells through its ability to bind to vitronectin. This promotes endothelial cells detachment from vitronectin and induces migration towards a fibronectin-rich and less vascularized tumor stroma. Due to its anti-fibrinolytic activity, PAI-1 increases fibrin deposition leading to endothelial cell organization and the release of angiogenic proteins such as interleukin (IL)-8 [31]. Studies have shown that PAI-1 promotes tumor cell migration by preventing the adhesion of cancer cells to vitronectin, which stimulates their migration toward other ECM substrates such as fibronectin. Importantly, by inhibiting uPAR-bound uPA, PAI-1 prevents excessive pericellular degradation of the ECM proteins that are necessary for cancer cell adhesion and migration [31].

A surprising result was the hypoxic time-dependent down-regulation of *PLAU* that encodes for the main plasminogen activator, uPA, in UP-007, UP-029, SEBTA-003, and U87 cells (Figure 5A–E). This result was also corroborated at the protein level in UP-029 and U87 cells, whereas there were no significant changes in the levels of uPA in the remaining GB cells during hypoxia (Figure 7). Several studies have shown that uPA plays a major role in the activation of plasmin and subsequent ECM degradation at the tumor site [8]. Studies have shown enhanced expression and activity of uPA in GB compared to LGG and normal brain tissue [34,35,36]. Importantly, high uPA levels in high grade gliomas (HGG), including GB, were associated with poor prognosis [34,36]. Our results showed either a decrease or no significant change in uPA levels in hypoxic GB cells compared to their normoxic controls, suggesting that uPA produced by cells within the tumor microenvironment might play a critical role in uPA/plasmin dependent ECM degradation in GB during hypoxia. Several studies have shown that the receptor for uPA, uPAR is up-regulated in GB and plays an important role in GB migration and invasion [37,38,39,40]. A previous report showed that HIF-1α promotes invasion by regulating the expression of *MMP-2* and *uPAR* in HCT116 colon carcinoma cells [41]. In contrast, we did not observe transcriptional regulation of *uPAR* in the GB cell lines investigated during hypoxia (Figure 5 and Figure S4). Nevertheless, uPAR protein levels were up-regulated during hypoxia in all GB cell lines investigated, except in UP-007 cells (Figure 7).

We also identified the differential hypoxia dependent expression of a number of genes and corresponding proteins with varied functions in our GB cells. We observed a significant up-regulation of *ADM* in all GB cells investigated during hypoxia. This result corroborates with previous reports showing up-regulation of *ADM* in hypoxic GB cells [42,43]. ADM is a 52 amino-acid secreted protein with a potent vasodilatory action and pro-angiogenic effect [44,45]. Hypoxic up-regulation of this protein in GB might therefore promote tumor angiogenesis. ADM also functions as a neuromodulator, promotes natriuretic and diuretic effects, regulates blood pressure and bronchodilation, inhibits proliferation and suppresses apoptosis [46]. Clinical data analysis showed a significant up-regulation of *ADM* expression in GB patient samples compared to non-neoplastic brain tissue (Figure 8A). Importantly, *ADM* expression was slightly lower in LGG compared to normal brain tissue, indicating that it could be used as a specific marker for GB (Figure S6). Moreover, high expression of *ADM* in GB was associated with worse overall survival (Figure 8K). Together these data indicate that ADM might constitute a promising target for GB therapy.

Our study also showed *ANGPTL4* up-regulation during hypoxia in all GB cell lines investigated, except in SEBTA-023 cells. Clinical data analysis further showed that *ANGPTL4* is highly expressed in GB, but not in LGG compared to normal brain tissue (Figure 8A and Figure S6). *ANGPTL4* expression was highest in GB hypoxic core region, but it was still significantly higher in the GB invasive margin compared to normal brain tissue (Figure 8B). ANGPTL4 is a secreted multifunctional protein that has been recognized as a central player in various aspects of energy homoeostasis, at least in part, via the inhibitory interaction between the coiled-coil domain of ANGPTL4 and lipoprotein lipase. ANGPTL4 fibrinogen-like domain interacts with and activates specific integrins to facilitate wound healing, modulation of vascular permeability, regulation of ROS levels, tumor growth, angiogenesis and invasion/metastasis [47,48,49,50]. A recent study showed that ANGPTL4 induces temozolomide resistance in U87 and Pt#3 GB cell lines by promoting glioma stem-like cells enrichment via the EGFR/AKT/4E-BP1 signaling pathway [51]. Another study showed that EGFRvIII induces c-myc mediated expression of ANGPTL4 in LN229 cells leading to GB growth and angiogenesis [52]. These studies together with our data demonstrating a highly significant up-regulation of *ANGPTL4* in GB cells during hypoxia and in GB patient samples, support that ANGPTL4 might constitute an important target for GB therapy, especially taking into account its contribution to many important features of GB progression, such as energy homeostasis, angiogenesis, invasion and chemoresistance.

We detected a significant up-regulation of *BNIP-3* during hypoxia in all GB cells investigated (Figure 6). BNIP-3 has been shown to inhibit apoptosis in GB cells by acting as a transcriptional repressor of the death receptor-5 expression and therefore preventing TRAIL-induced cell death in gliomas [53,54]. BNIP-3 has also been shown to trigger selective mitochondrial autophagy (mitophagy) contributing to GB cell survival during hypoxia [55]. Hypoxia induces mitochondrial dysfunction and consequently leakage of ROS from this organelle to the cytoplasm. Mitophagy is therefore an important mechanism during hypoxia to avoid the deleterious effects of high levels of intracellular ROS on proteins, lipids and DNA which could lead to cell death. In addition, the degradation products of these mitochondrion can also be recycled and used in the biosynthesis of cellular components and in the production of energy. However, clinical data analysis showed a modest up-regulation of *BNIP-3* in GB compared to normal brain tissue (Figure 8A), and we did not observe association of this gene with patient overall survival (data not shown).

*DDIT4* was highly over-expressed (10–20 fold) in all GB cell lines investigated during hypoxia (Figure 6). *DDIT4* over-expression in U87FO and U373FO GB cell lines during hypoxia had been previously reported [56]. Clinical data analysis showed high expression of *DDIT4* in GB patient samples, with *DDIT4* highest levels in GB hypoxic core region but also significantly higher in GB invasive margin compared to normal brain tissue (Figure 8A,B). The *DDIT4* gene encodes for the REDD1 protein which activates the mTORC1 negative regulator, Tuberous Sclerosis 1/2 (TSC1/TSC2) complex [57]. mTORC1 multiprotein complex constitutes a major regulator of cell growth, translation, and metabolism, and has also been shown to inhibit autophagy [58,59]. Interestingly, over-expression of REDD1 in GB has been associated with resistance to temozolomide, radiotherapy, and resistance to hypoxia induced cell death, as well as linked to patient poor prognosis [60,61]. Nevertheless, the molecular mechanism(s) by which REDD1 promotes GB progression are still poorly understood. One possible explanation would be the induction of autophagy via mTOR inhibition, which could lead to the recycling of cellular components and subsequent survival under hypoxic conditions, where restriction of nutrients and lack of oxygen for energy production are observed. Further studies are necessary to investigate this hypothesis.

We observed a highly significant hypoxic up-regulation of *NDRG1* (from 20 up to 240 fold depending on the GB cell line analyzed) which correlated with enhanced NDRG1 protein expression in all GB cell lines investigated (Figure 6 and Figure 7). These data support previous reports showing NDRG1 up-regulation in hypoxic GB [62,63]. *NDRG1* was highly expressed in GB but not in LGG clinical samples compared to normal brain tissue (Figure 8A and Figure S6). NDRG1 is a multifaceted protein that has been shown to regulate nerve myelination, stress response, lipid biosynthesis and metabolism, exocytosis and differentiation [64]. NDRG1 has been shown to confer resistance to temozolomide, via binding and stabilization of MGMT [65]. This report in conjunction with the fact that hypoxia promotes a potent induction of NDRG1 indicate that this protein might constitute a promising target for GB therapy.

Hypoxia induced *EGR1* down-regulation in most GB cell lines examined, with the exception of UP-029 cells where we observed a transient down-regulation of *EGR1* at 6 h and 24 h of hypoxia followed by a 3-fold induction at 48h of hypoxia. Previous research works have shown contradictory results regarding the hypoxic regulation of *EGR1* in GB cells. While Said et al. reported no changes in *EGR1* mRNA levels during hypoxia in GaMG, U87, U373 and U251 GB cells [66]. Rong et al. stated that hypoxia induced *EGR1* expression in U87 cells [67]. EGR1 is a Cys2-His2-type zinc finger transcription factor that in GB cell lines has been shown to bind to GC-rich sites of the TGF-β promotor leading to decreased proliferation and suppressing transformation [68]. In contrast, another study showed that EGR1 induces U251 cell migration via activation of fibronectin [69]; and other reports indicated that EGR1 confers temozolomide resistance in GB [70,71]. EGR1 has also been shown to promote a glioma stem cell-like phenotype [72,73]. In summary, the role of EGR1 in GB seems complex and most likely depends on the specific stimuli, cell line specificity and microenvironmental conditions.

Analysis of *TFRC* expression showed down-regulation of this gene in UP-029 cells, a transient down-regulation at 6 h and/or 24 h of hypoxia in UP-007, SEBTA-023 and U87 cells and no changes in hypoxic SEBTA-003 cells (Figure 6). *TFRC* hypoxic regulation in GB cells has not been previously reported. We observed a hypoxia time-dependent down-regulation of TFRC protein in UP-007 and U87 cells, but not in the remaining GB cells (Figure 7). Together, these data suggest that hypoxic regulation of TFRC in GB is cell line dependent. Further studies are necessary to fully characterize the molecular mechanism(s) involved in TFRC hypoxic regulation in GB cells. Clinical data analysis showed significantly higher expression of *TFRC* in GB, but not LGG patient samples compared to normal tissue (Figure 8A and Figure S6). TFRC is a membrane glycoprotein, which is involved in the import of iron by binding to the plasma glycoprotein, transferrin (TF) that possesses two specific Fe (III) binding sites [74]. TFRC is abnormally expressed in many different types of cancer and has been associated with cancer cell proliferation, apoptosis, migration and invasion/metastasis. However, the molecular mechanisms by which TFRC contributes to tumor progression remain elusive [74]. A previous report showed association of *TFRC* over-expression with worse overall survival in GB patients [75]. Analysis of our clinical data further supported this report (Figure 8J). It was therefore interesting to observe a hypoxia dependent down-regulation of TFRC in two GB cells lines (UP-007, U87). We hypothesize that this could be important for inhibition of PHDs, the negative regulators of HIF-α, whose function is dependent on Fe (III). In fact, we did not observe up-regulation of HIF-1α protein levels in hypoxic U87 cells compared to their normoxic control cells, even though we observed up-regulation of many HIF-1α target genes in this GB cell line. These results indicate that there are additional regulatory mechanisms involved in the up-regulation of HIF-1 function in the U87 cells during hypoxia, that are independent of HIF-1α levels. Further studies are necessary to investigate this hypothesis.

## 5. Conclusions

In this work we characterized the hypoxic response of GB cells using 4 biopsy patient derived cell lines and the widely used U87 cell line. Overall, our data showed that the majority of genes and proteins regulated by hypoxia were conserved across all GB cells investigated. Unsurprisingly, the U87 cell line showed the highest disparity compared to the biopsy derived GB cells, which is probably due to the fact that this cell line has been in culture for many decades and as such likely differs from the original GB cells. Taking into account that GBs are highly heterogeneous tumors and the fact that hypoxia is greatly associated with GB mortality and chemoresistance, these data provide encouraging GB molecular markers and targets for the potential development of novel and effective therapies for GB patients in the future.

In summary, we observed a hypoxia triggered metabolic switch towards glycolysis where the genes/proteins, HK2, PFKFB3, PFKFB4, LDHA, PDK1, *SLC2A1*/GLUT-1, *CA9*/CA IX, and *SLC16A3*/MCT-4 were significantly up-regulated in all GB cell lines (Graphical Abstract). We observed the hypoxic up-regulation of many pro-angiogenic genes and proteins, namely VEGFA, VEGFC, VEGFD, *PGF*/PlGF, *ADM*, *ANGPTL4*, and *SERPINE1/*PAI-1 (Graphical Abstract). While some of the hypoxia induced angiogenic responses were conserved among all GB cells investigated (e.g., VEGFA; VEGFC, VEGFD, *ADM*), others were cell line specific (e.g., *PGF*/PlGF, *SERPINE1*/PAI-1, *ANGPTL4*). These data show the complexity and potency of the GB angiogenic response to hypoxia, indicating that targeting GB angiogenesis is most likely a multifarious task. GB is a highly invasive tumor, interestingly we found that hypoxia did not induce *MMP-2*, *MMP-9*, *PLAU*, *uPAR, CTSA*, or *ANXA2* gene transcription in our GB cell models. Importantly, we observed hypoxia-dependent induction of proteins that constitute the plasminogen system, namely PAI-1, the plasminogen receptor, S100A10, and the receptor for the uPA, uPAR. S100A10 and uPAR co-localize at the cell surface bringing together plasminogen and uPA and therefore promoting the generation of the serine protease, plasmin and subsequent degradation of the ECM which is fundamental for cancer cell invasion. Plasmin is also able to cleave and activate many MMPs, further exacerbating ECM degradation (Graphical Abstract). This is the first study showing hypoxia dependent up-regulation of *S100A10* mRNA and protein levels in GB. We showed hypoxic up-regulation of genes involved in promoting autophagy, such as *BNIP-3* and *DDIT4*. Autophagy has been shown to have a cytoprotective role during hypoxia, allowing the recycling of cellular components under nutrient and oxygen restrictive conditions. Furthermore, we observed hypoxic up-regulation of genes and proteins that provide GB chemoresistance, namely *ANGPTL4*, *DDIT4* and NDRG1.

In conclusion, this study analyses GB response to hypoxia incorporating in vitro patient derived GB cell lines and ex vivo clinical data. Our data identified potential molecular markers and targets for GB therapy that are involved in the regulation of key aspects of GB tumourigenesis, such as cancer cell metabolism, angiogenesis, invasion and therapy resistance.

## Supplementary Materials

The following are available online at https://www.mdpi.com/2227-9059/8/9/310/s1, Figure S1: Relative expression of transcription factors and glycolysis related genes compared to *RPLP0*; Figure S2: Relative expression of hypoxic related genes as compared to *RPLP0*; Figure S3: *S100A10* expression in hypoxic SEBTA-023 cells compared to normoxic control cells; Figure S4: *MMP-2* and *MMP-9* expression in hypoxic GB cells compared to normoxic control cells; Figure S5: Protein expression analysis in non-neoplastic astrocytes and a panel of cancer cell lines; Figure S6: Gene expression in low grade and high grade gliomas compared to non-neoplastic brain tissue; Figure S7: Protein expression analysis in hypoxic versus normoxic GB cells; Table S1: Primary glioblastoma cell lines molecular characterization; Table S2: Antibodies list; Table S3: RT-qPCR primers list; Table S4: Gene expression analysis in hypoxic GB cells compared to their normoxic controls; Table S5: RT-qPCR gene fold-change analysis in GB cells subjected to 6 h, 24 h, or 48 h of hypoxia compared to normoxic control cells; Supplementary Materials and Methods.
