# Supplementary material for: Investigating Glioblastoma Response to Hypoxia
Source: Biomedicines. 2020 Aug 27;8(9):310. doi: 10.3390/biomedicines8090310 (PMC7555589; doi:10.3390/biomedicines8090310)
Supplement: Supplementary file 1 [file biomedicines-08-00310-s001.zip › Table S5.pdf]

Table S5. RT-qPCR gene fold-change analysis in GB cells subjected to 6h, 24h or 48h of hypoxia compared to normoxic control cells.

	UP-007			UP-029			SEBTA-003			SEBTA-023			U87		
**Hypoxia**	**6h**	**24h**	**48h**	**6h**	**24h**	**48h**	**6h**	**24h**	**48h**	**6h**	**24h**	**48h**	**6h**	**24h**	**48h**
HIF-1α	0.9±0.1	0.5±0.02	0.3±0.08	0.8±0.02	0.5±0.01	1±0.2	0.9±0.04	0.6±0.02	0.5±0.03	1.2±0.2	1.7±0.3	1.3±0.2	0.9±0.03	0.7±0.06	1±0.3
HIF-2α	1.3±0.1	2.9±0.8	3.3±0.3	2.3±0.2	10.5±2.2	12.9±3	1.2±0.05	1.4±0.3	1.2±0.1	1.5±0.2	2±0.4	4.2±1.5	1±0.1	1±0.2	1.1±0.1
Nrf2	0.6±0.1	0.6±0.1	0.7±0.4	1.1±0.3	0.7±0.2	1.3±0.4	ND	ND	ND	1±0.2	1.1±0.4	1.2±0.4	0.7±0.3	0.6±0.3	0.7±0.3
SLC2A1	1.6±0.1	3.2±0.4	5.5±0.7	5.1±0.6	9.9±2.3	9.3±0.9	5.8±0.6	11.5±0.4	13.4±1	2.4±0.2	6±0.8	4.5±0.6	8.9±1.1	17.5±2.6	16.1±1
LDHA	1.6±0.07	2.9±0.6	2.9±1.1	2±0.2	3.3±0.2	2.6±0.2	1.6±0.2	3.3±0.2	2.6±0.01	3±0.7	7.2±0.7	9±1.4	2.1±0.08	4.4±0.2	4±0.4
PDK1	3.9±0.8	8.4±2.6	8.6±1.4	5.2±0.9	9.3±2	7.2±1	3±0.1	4.2±0.2	3.2±0.1	5.4±1	6.4±1.6	7.3±1.3	6.2±0.4	15.2±1.3	9±0.9
PFKFB3	3.1±0.7	4.6±1.5	4.2±0.9	7.6±1	9.9±0.6	7.7±2.1	2.4±0.3	2.2±0.3	2.6±0.4	5±0.9	2.1±0.1	4.6±1.1	4.8±0.7	4.6±1	7.1±0.9
PFKFB4	3.3±0.8	4.2±1.1	3.8±1	5.3±1	5.2±1.4	3.3±0.2	4.7±1.1	5.8±0.7	4.6±0.8	5.2±1.8	9.4±2.9	3.6±0.3	4.1±0.1	5.7±0.4	3.4±0.2
SLC16A3	1.4±0.1	1.7±0.2	2±0.5	6±0.5	9.8±1.4	10.2±2	5.1±1	9.2±1.2	4.7±0.9	4.8±0.9	13.2±3.2	15.7±2.8	2.9±0.4	2.3±0.2	2.4±0.5
HK2	5.1±0.04	6±0.2	6.3±0.5	5.7±0.4	6.5±0.9	4.4±0.6	3.4±0.09	4.2±0.1	3.3±0.4	30.6±3.4	23.9±5.3	26±1	4.5±0.5	9.3±1.3	10±0.9
CA9	2.7±0.3	9.1±1.8	11±2.1	5.7±0.8	29.2±5	33.9±7.8	14.9±0.7	15±1.2	20.5±4.5	5.7±0.8	39±8.5	59.6±5.6	3±0.2	15.2±1.4	13.9±1.7
VEGFA	1.3±0.1	4.3±0.9	6.6±1.1	13.4±3.4	39.5±6.4	40.7±10	2.9±0.2	2.9±0.05	2.6±0.2	3±0.4	10.8±1.5	5.7±1.5	3.8±0.8	5±1.2	5.3±1.5
VEGFC	0.9±0.1	0.9±0.05	1.1±0.1	0.8±0.2	0.8±0.03	0.9±0.2	1.1±0.05	0.9±0.07	0.8±0.03	1.1±0.3	1.3±0.4	2.7±0.7	0.9±0.1	0.7±0.2	1±0.3
VEGFD	0.8±0.09	1.3±0.3	1.7±0.3	1±0.2	1.4±0.3	1.7±0.4	1±0.02	1.3±0.3	1.4±0.04	0.9±0.3	1.2±0.2	0.9±0.3	1.2±0.03	1.4±0.2	1.2±0.2
PGF	0.8±0.1	1.1±0.1	1.3±0.2	5.6±0.3	32.2±8.3	4.8±0.2	5.4±0.8	15.3±1.4	4.7±0.4	1±0.09	0.9±0.08	1.3±0.1	0.9±0.1	1.3±0.2	1.6±0.2
ANXA2	0.9±0.2	1.1±0.2	1.1±0.2	1±0.06	1.1±0.3	1.4±0.4	1±0.06	1.8±0.06	1.4±0.1	0.9±0.2	0.7±0.09	0.7±0.08	1.1±0.1	1.1±0.1	1±0.1
S100A10	0.9±0.07	1.3±0.2	2.2±0.09	1.4±0.2	2.6±0.08	1.9±0.2	1.2±0.1	2.1±0.4	1.7±0.3	1.3±0.3	2±0.5	1.9±0.3	1.1±0.1	1.8±0.08	1.2±0.2
PLAU	0.4±0.02	0.3±0.01	0.2±0.02	0.55±0.2	0.3±0.05	0.3±0.09	0.5±0.06	0.1±0.01	0.04±0.007	1±0.3	1±0.08	1.3±0.4	0.8±0.1	0.2±0.01	0.2±0.03
uPAR	0.7±0.04	0.6±0.08	0.7±0.08	1.1±0.05	1±0.2	1.1±0.3	1.2±0.2	1.2±0.1	0.6±0.06	1.3±0.5	1.4±0.3	1.3±0.1	1.2±0.3	1.2±0.1	1.1±0.3
SERPINE1	1.2±0.1	1.8±0.3	1.1±0.1	1.1±0.3	1.1±0.3	1.2±0.6	3.3±0.7	5.7±0.8	2.2±0.3	1.2±0.5	1.4±0.1	2±0.2	4.5±0.5	4.6±0.4	5.7±0.6
MMP-2	1±0.3	1.1±0.1	1.1±0.2	0.7±0.08	2±0.4	3.8±1.7	NDT	NDT	NDT	1.2±0.4	1.2±0.5	1.3±0.4	0.9±0.04	1.1±0.08	1.4±0.1
MMP-9	1.2±0.06	0.9±0.1	1±0.09	0.7±0.2	0.9±0.3	0.9±0.4	1±0.02	1.8±0.1	1±0.2	0.8±0.1	0.4±0.1	0.4±0.1	0.55±0.1	0.5±0.03	0.9±0.3
DDIT4	10.7±2.2	7.5±0.3	9.6±2.1	18.7±1.4	38.2±3	17.5±2.5	8.9±1.2	6.2±0.7	4.5±0.7	12.9±2.1	19.4±5.5	16.3±4.1	9.5±1.6	7.5±1.2	11.7±2.6
NDRG1	2.6±0.6	11.6±2.7	18.3±6	21.3±1.5	172±9	242±66	7.2±0.8	23.7±1.5	21±2.2	2.7±0.7	37.7±3.7	53.9±4.6	12.6±3.9	24.2±2	29.2±3.4
BNIP3	2.3±0.5	6.9±2.5	2.5±0.3	3.7±0.4	4.4±0.5	9.5±4	3.3±0.4	7±0.4	6.4±0.5	5.2±0.1	14.2±4.1	10±1.5	4.1±0.1	12.4±0.8	11.3±1.1
ANGPTL4	1.6±0.2	4.8±1.1	3.9±0.8	2.7±0.3	12.6±0.6	6.1±0.5	8.3±2.7	5.6±2.3	4±2.3	1.2±0.06	1±0.2	1.2±0.2	2.8±0.02	3.2±0.4	3.5±0.2
ADM	3±0.8	5.3±0.8	5.6±1	5.6±1.1	8±2.5	5±0.8	2.7±0.3	2.2±0.2	2±0.2	15.4±3.9	7.5±1	5.8±2	3.2±0.6	4.6±0.4	6.8±1.8
EGR1	0.3±0.07	0.4±0.08	0.5±0.08	0.6±0.1	0.3±0.06	2.9±0.6	0.8±0.06	0.4±0.02	0.5±0.05	1±0.1	0.5±0.3	0.5±0.2	0.4±0.07	0.3±0.01	0.5±0.02
TFRC	0.3±0.04	0.5±0.07	1.8±0.2	0.6±0.03	1.1±0.07	0.3±0.02	1.1±0.1	1±0.1	1.1±0.1	0.4±0.1	0.9±0.05	0.8±0.09	0.3±0.1	0.3±0.06	1±0.15
UCP2	1±0.05	1.2±0.1	0.6±0.03	0.9±0.03	0.5±0.1	0.3±0.02	0.9±0.15	0.4±0.04	0.2±0.02	1.1±0.1	0.5±0.09	0.5±0.08	1.2±0.2	0.6±0.08	0.3±0.04

ND-not determined; NDT-not detected
