# Supplementary material for: Investigating Glioblastoma Response to Hypoxia
Source: Biomedicines. 2020 Aug 27;8(9):310. doi: 10.3390/biomedicines8090310 (PMC7555589; doi:10.3390/biomedicines8090310)
Supplement: Supplementary file 1 [file biomedicines-08-00310-s001.zip › Figure S2.pdf]

Figure S2

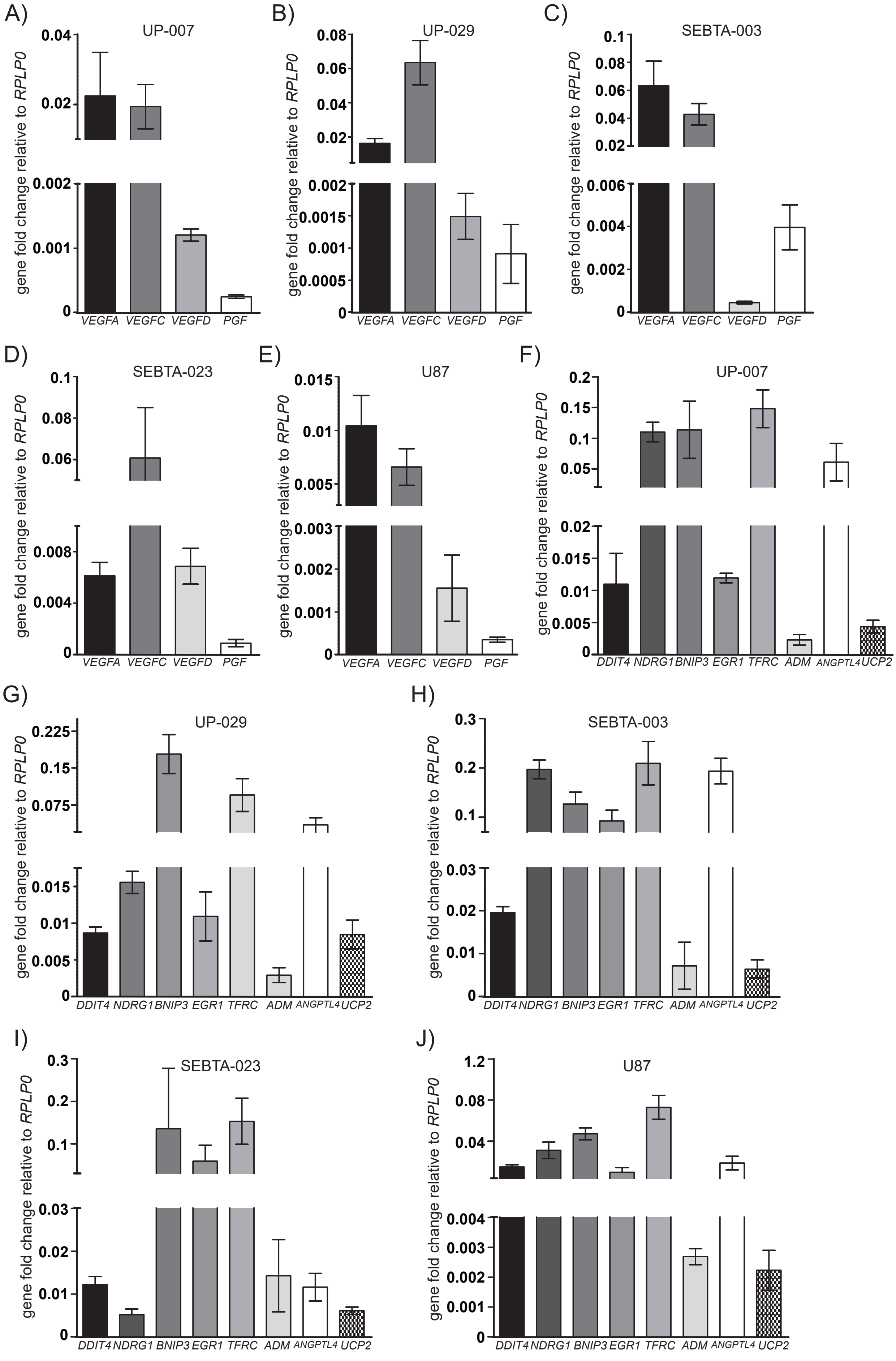
A)
UP-007
B)
UP-029
C)
SEBTA-003
D)
SEBTA-023
E)
U87
F)
UP-007
G)
UP-029
H)
SEBTA-003
I)
SEBTA-023
J)
U87
gene fold change relative to RPLP0
VEGFA VEGFC VEGFD PGF
DDIT4 NDRG1 BNIP3 EGR1 TFRC ADM ANGPTL4 UCP2
