# Supplementary material for: Investigating Glioblastoma Response to Hypoxia
Source: Biomedicines. 2020 Aug 27;8(9):310. doi: 10.3390/biomedicines8090310 (PMC7555589; doi:10.3390/biomedicines8090310)
Supplement: Supplementary file 1 [file biomedicines-08-00310-s001.zip › Supplementary Figure legends.pdf]

Figure S1. Relative expression of transcription factors and glycolisis related genes compared to *RPLP0*. Total RNA of normoxic (A,F) UP-007; (B,G) UP-029; (C,H) SEBTA-003; (D,I) SEBTA-023 or (E,J) U87 cells was extracted using the NZY Total RNA Isolation kit (Nzytech, Portugal) according to the manufacturer´s instructions. The gene expression was determined by RT-qPCR using the One-step NZYSpeedy RT-qPCR Green kit (Nzytech, Portugal) according to the manufacturer´s instructions. The reaction was carried out using the LightCycler 96 instrument (Roche). Relative fold change was calculated by applying the following formula $2^{\wedge-\Delta Ct}$, where ΔCt is obtained by subtracting *RPLP0* Ct value to the Ct value of each gene of interest, as indicated. Error bars represent the Standard Deviations obtained from at least three independent experiments.

Figure S2. Relative expression of hypoxic related genes compared to *RPLP0*. Total RNA of normoxic (A,F) UP-007; (B,G) UP-029; (C,H) SEBTA-003; (D,I) SEBTA-023 or (E,J) U87 cells was extracted using the NZY Total RNA Isolation kit (Nzytech, Portugal) according to the manufacturer´s instructions. The gene expression was determined by RT-qPCR using the One-step NZYSpeedy RT-qPCR Green kit (Nzytech, Portugal) according to the manufacturer´s instructions. The reaction was carried out using the LightCycler 96 instrument (Roche). Relative fold change was calculated by applying the following formula $2^{\wedge-\Delta Ct}$, where ΔCt is obtained by subtracting RPLP0 Ct value to the Ct value of each gene of interest, as indicated. Error bars represent the Standard Deviations obtained from at least three independent experiments.

Figure S3. *S100A10* expression in hypoxic SEBTA-023 cells compared to normoxic control cells. SEBTA-023 cells were incubated under normoxia (21% O_2) or hypoxia (1% O_2) for the times indicated. After what RNA extraction was performed using the NZY Total RNA Isolation kit (Nzytech, Portugal) according to the manufacturer´s instructions. The gene expression was determined by RT-qPCR using the One-step NZYSpeedy RT-qPCR Green kit (Nzytech, Portugal) according to the manufacturer´s instructions. The reaction was carried out using the LightCycler 96 instrument (Roche). Gene expression levels were normalized to *RPLP0* mRNA using the $2^{-\Delta\Delta CT}$ method (Livak KJ, Schmittgen TD). Error bars represent the Standard Deviations obtained from at least three independent experiments. Statistical analysis was evaluated using two-tailed Student's t-test, comparing each hypoxia time-point to the respective normoxic control. In every case a P value of less than 0.05 (*), less than 0.01(**) and 0.001 (***) was considered statistically significant.

Figure S4. *MMP-2* and *MMP-9* expression in hypoxic GB cells compared to normoxic control cells. (A) UP-007; (B) UP-029; (C) SEBTA-003; (D) SEBTA-023 or (E) U87cells were incubated under normoxia (21% O_2) or hypoxia (1% O_2) for the times indicated. After what RNA extraction was performed using the NZY Total RNA Isolation kit (Nzytech, Portugal) according to the manufacturer´s instructions. The gene expression was determined by RT-qPCR using the One-step NZYSpeedy RT-qPCR Green kit (Nzytech, Portugal) according to the manufacturer´s instructions. The reaction was carried out using the LightCycler 96 instrument (Roche). Gene expression levels were normalized to *RPLP0* mRNA using the $2^{-\Delta\Delta CT}$ method (Livak KJ, Schmittgen TD). Error bars represent the Standard Deviations obtained from at least three independent experiments. Statistical analysis was evaluated using two-tailed Student's t-test, comparing each hypoxia time-point to the respective normoxic control. In every case a P value of less than 0.05 (*), less than 0.01(**) and 0.001 (***) was considered statistically significant.

Figure S5. Protein expression analysis in SC-1800 non-neoplastic astrocytes and a panel of cancer cell lines. SC-1800, non-neoplastic astrocytes; SEBTA-023, UP-029, SEBTA-003, UP-007, SEBTA-025, U87 GB cell lines; and MDA-MB-231 breast carcinoma cells were incubated under normoxic (21% O_2) conditions. After what cells were lysed and 20 μg of each protein extract was subjected

to SDS-PAGE, transferred onto nitrocellulose membranes and analyzed by western blotting with the antibodies indicated.

Figure S6. Gene expression in low grade and high grade gliomas compared to non-neoplastic brain tissue. Expression of selected hypoxia-related genes in normal brain, Low Grade Gliomas (LGG) and GB patient samples. Data sourced from the published study with gene expression microarrays generated from 7 normal brain and 217 GB tumours. Normalized expression values on $\log_2$ scale are indicated on the y-axis. In the boxplots, the top, middle and bottom box delimiters represent the 75th, 50th and 25th percentiles of the data, respectively. Top and bottom whiskers show the 75th percentile + 1.5*interquartile range and 25th percentile – 1.5*interquartile range, respectively.

Figure S7. Protein expression analysis in hypoxic versus normoxic GB cells. UP-029 and SEBTA-023 cells were incubated under normoxic (21% O_2) or hypoxic (1% O_2) conditions for the times indicated. After what cells were lysed and 20 μg of each protein extract was subjected to SDS-PAGE, transferred onto nitrocellulose membranes and analyzed by western blotting with the antibodies indicated.
