# Supplementary material for: Investigating Glioblastoma Response to Hypoxia
Source: Biomedicines. 2020 Aug 27;8(9):310. doi: 10.3390/biomedicines8090310 (PMC7555589; doi:10.3390/biomedicines8090310)
Supplement: Supplementary file 1 [file biomedicines-08-00310-s001.zip › Supplementary Materials and Methods.pdf]

Supplementary Materials and Methods:

Cell lines and cell culture

Human adult non-neoplastic astrocytes, SC-1800 were obtained from ScienCell Research Laboratories. SC-1800 cells were grown in AGM Astrocyte Growth Medium BulletKit (LONZA). MDA-MB-231 cell line was obtained from ATCC. MDA-MB-231 cells were grown in DMEM (HyClone™) supplemented with 10% fetal bovine serum (FBS), 20 mM L-Glutamine. Cells were maintained in a humidified incubator with 21% O_2 at 37˚C with 5% CO_2. Cells were tested for mycoplasma contamination regularly.
