# Supplementary material for: Investigating Glioblastoma Response to Hypoxia
Source: Biomedicines. 2020 Aug 27;8(9):310. doi: 10.3390/biomedicines8090310 (PMC7555589; doi:10.3390/biomedicines8090310)
Supplement: Supplementary file 1 [file biomedicines-08-00310-s001.zip › Table S1.pdf]

Table S1. Primary glioblastoma cell lines molecular characterization.

Cell line	MGMT promoter status	IDH status
UP-007	methylated	WT
UP-029	methylated	WT
SEBTA-003	methylated	WT
SEBTA-023	methylated	WT
