# Supplementary material for: Investigating Glioblastoma Response to Hypoxia
Source: Biomedicines. 2020 Aug 27;8(9):310. doi: 10.3390/biomedicines8090310 (PMC7555589; doi:10.3390/biomedicines8090310)
Supplement: Supplementary file 1 [file biomedicines-08-00310-s001.zip › Table S2.pdf]

Table S2. Antibodies list.

Target protein	reference	Company
ANXA2 (D1/274.5)	sc-65873	Santa Cruz Biotechnology (SCBT)
S100A10 (4E7E10)	sc-81153	SCBT
GLUT-1 (A4)	sc-377228	SCBT
uPA	sc-59727	SCBT
uPAR	PA1344	BosterBio
PAI-1 (C-9)	sc-5297	SCBT
PFKFB3 (D-1)	sc-377416	SCBT
Aldolase A	sc-12059	SCBT
HIF-1α (28B)	sc-13515	SCBT
HIF-2α (A-5)	sc-46691	SCBT
HK2 (B-8)	sc-374091	SCBT
LDHA (E-9)	sc-137243	SCBT
PDK1 (4A11F5)	sc-293160	SCBT
CA IX (H-11)	sc-365900	SCBT
VEGFA (C-1)	sc-7269	SCBT
VEGFC (E-6)	sc-374628	SCBT
VEGFD	sc-13085	SCBT
PlGF (H-4)	sc-518003	SCBT
TFRC/ CD71 (286)	sc-51829;	SCBT
NDRG1 (B-5)	sc-398291	SCBT
UCP2 (G-6)	sc-390189	SCBT
P-AKT Ser 473	sc-7985-R	SCBT
AKT	9272	SCBT
P-ERK 1/2	sc-16982	SCBT
ERK 1/2 (MK1)	sc-135900	SCBT
Cyclophilin B	Ab16045	AbCam
β-tubulin	sc-5286	SCBT
actin (C-11)	sc-1615	SCBT
GAPDH (FL-335)	sc-25778	SCBT
IRDye 800 CW Anti-mouse 788	926-32210	LI-COR
IRDye 800 CW Anti-rabbit 788	926-32211	LI-COR
