# Supplementary material for: Investigating Glioblastoma Response to Hypoxia
Source: Biomedicines. 2020 Aug 27;8(9):310. doi: 10.3390/biomedicines8090310 (PMC7555589; doi:10.3390/biomedicines8090310)
Supplement: Supplementary file 1 [file biomedicines-08-00310-s001.zip › Table S3.pdf]

Table S3. RT-qPCR primers list.

Gene	Forward primer	Reverse primer
ADM	TGA AGC TGG TTT CCG TCG	AGA GCC CAC TTA TTC CAC TTC
ANGPTL4	TCT CTG GAG GCT GGT GGT TT	AGA GTC ACC GTC TTT CGT GG
ANXA2	CTC TAC ACC CCC AAG TGC AT	TCA GTG CTG ATG CAA GTT CC
BNIP3	CGC AGA CAC CAC AAG ATA CCA AC	GCC AGC AAA TGA GAG AGC AGC
CAIX	TTT GCC AGA GTT GAC GAG G	AGC CTT CCT CAG CGA TTT C
DDIT4	GAC AGC AGC AAC AGT GGC TTC G	GCT GCA TCA GGT TGG CAC AC
EGR1	ACC GCA GAG TCT TTT CCT GAC A	GGT GCA GGC TCC AGG GAA AA
HIF-1α	ATC CAT GTG ACC ATG AGG AAA TG	TCG GCT AGT TAG GGT ACA CTT C
HIF-2α	GTG CCA TGA CAA ACA TCT TCC AG	CTC GGG CTC TGT CTT CTT GCT
HK2	TCA CGG AGC TCA ACC ATG AC	CCC AAA GCA CAC GGA AGT TG
LDHA	GCT GG TCA TTA TCA CGG CTG	AGC AAC TTG CAG TTC GGG CTG
MMP-2	ACC CAT TTA CAC CTA CAC CAA G	TGT TTG CAG ATC TCA GGA GTG
MMP-9	CGA ACT TTG ACA GCG ACA AG	CAC TGA GGA ATG ATC TAA GCC C
NDRG1	CTG CAC CTG TTC ATC AAT GC	AGA GAA GTG ACG CTG GAA CC
Nrf2	TCA TGA TGG ACT TGG AGC TG	CAT ACT CTT TCC GTC GCT GA
PDK1	CTG TGA TAC GGA TCA GAA ACC G	TCC ACC AAA CAA TAA AGA GTG CT
PFKFB3	ATC TAC CTG AAC GTG GAG TCC GTC TG	TCA GTG TTT CCT GGA GGA GTC AGC
PFKFB4	TTA ATT TTG GAG AAC AGA ATG GC	CGT AGC CTC ATC ACT GTC GC
PlGF	TGC GGC GAT GAG AAT CTG C	AGC GAA CGT GCT GAG AGA AC
uPA	CAG GGC ATC TCC TGT GCA TG	AGC CCT GCC CTG AAG TCG TTA
uPAR	GCC TTA CCG AGG TTG TGT GT	CAT CCA GGC ACT GTT CTT CA
PAI-1	GGG CCA TGG AAC AAG GAT GA	CTC CTT TCC CAA GCA AGT TG
S100A10	AAA TTC GCT GGG GAT AAA GG	AGC CCA CTT TGC CAT CTC TA
SLC2A1	CTC CTG CCC TGT TGT GTA TAG	CAG GAG TGA GGT GGT GTA TTT
SLC16A3	TGT GTG CGT GAA CCG CTT T	AAA CCC AAC CCC GTG ATG AC
TFRC	ACT TGC CCA GAT GTT CTC AG	GTA TCC CTC TAG CCA TTC AGT G
vEGFA	AGT CCA ACA TCA CCA TGC AG	TTC CCT TTC CTC GAA CTG ATT T
vEGFC	GCC CCA AAC CAG TAA CAA TC	TCT TGT TCG CTG CCT GAC
vEGFD	ATG GAC CAG TGA AGC GAT CAT	GTT CCT CCA AAC TAG AAG CAG C
UCP2	AGT CCG GTT ACA GAT CCA AGG	AGC CCA TTG TAG AGG CTT CG
RPLP0	AGA CAA TGT GGG CTC CAA GCA GAT	GCA TCA TGG TGT TCT TGC CCA TCA

Note: all primer are represented with the orientation 5´ to 3´.
