# Supplementary material for: Investigating Glioblastoma Response to Hypoxia
Source: Biomedicines. 2020 Aug 27;8(9):310. doi: 10.3390/biomedicines8090310 (PMC7555589; doi:10.3390/biomedicines8090310)
Supplement: Supplementary file 1 [file biomedicines-08-00310-s001.zip › Table S4.pdf]

Table S4. Gene expression analysis in hypoxic GB cells compared to their normoxic controls. UP-029 and SEBTA-023 cells were either incubated under normoxic conditions (21% O_2) or under hypoxia (1% O_2) for the times indicated. RNA was extracted using the NZY Total RNA Isolation kit (Nzytech. Portugal) according to the manufacturer´s instructions. A panel of 86 ROS dependent genes was analysed using the Hypoxia Signaling Pathway RT2 Profiler PCR Array (QIAGEN) according to the manufacturer's instructions in a LightCycler 96 instrument (Roche). Results represent gene fold change in the hypoxia time-points indicated as compared to the respective normoxic control cells.

	Fold Change (compared to respective normoxic control)			
	SEBTA-023		UP-029	
Symbol	6h	48h	6h	48h
ADM	0.9794	2.4116	4.8568	2.3104
ADORA2B	0.9395	0.5249	1.0943	0.683
ALDOA	1.0867	1.1408	1.0497	1.4743
ANGPTL4	5.0982	3.7842	56.8859	9.1896
ANKRD37	2.6945	1.6358	4.3169	1.2834
ANXA2	0.9075	0.669	0.9395	0.6071
APEX1	0.6242	0.3842	0.6071	0.5105
ARNT	0.7526	0.9659	0.6113	0.8645
ATR	0.732	0.5	0.6926	0.5704
BHLHE40	1.879	1.1728	1.7532	0.9659
BLM	0.7071	0.4263	0.5471	1.2058
BNIP3	3.1167	4.7899	3.4581	2.6208
BNIP3L	1.5263	1.6702	1.815	2.4623
BTG1	0.9266	0.7474	1.6472	1.8404
CA9	15.6707	116.9704	11.5514	30.91
CCNG2	1.3287	0.8351	1.9725	1.9053
COPS5	0.683	0.4414	0.79	0.5864
CTSA	0.6926	0.5783	0.7071	1.8661
DDIT4	6.105	6.2767	5.8563	1.9588
DNAJC5	0.6974	0.5625	0.7526	0.7022
EDN1	0.8706	0.409	0.4897	0.7579
EGLN1	2.0705	1.2311	1.9588	1.6702
EGLN2	0.7631	0.5864	0.5743	0.2793
EGR1	0.7955	0.4569	0.2483	3.1602
EIF4EBP1	0.727	0.6552	0.933	0.717
ENO1	1.014	1.007	1.0425	0.5625
EPO	0.2398	2.1886	1.2746	2.0994
ERO1A	1.2397	1.6472	1.6358	0.712
F10	0.4796	0.933	0.3536	1.7411
F3	0.8011	0.6029	4.1411	0.4353
FOS	1.3379	0.5434	0.4175	1
GBE1	1.0644	1.5263	1.3947	0.683
GPI	1.0792	1.1487	1.0353	1.3947
GYS1	1.2142	1.057	1.6133	2.6945
HIF1A	0.6507	0.6329	0.4118	0.3686
HIF1AN	0.6736	0.5586	0.6417	0.6783
HIF3A	0.9794	2.4116	1.2058	1.9862
HK2	7.3615	4.6268	2.9282	1
HMOX1	0.9727	0.5704	0.9138	0.483
HNF4A	0.0183	0.2466	0.1497	0.0689
IER3	0.2912	1.2746	2.2974	0.4414
IGFBP3	0.8467	1.0281	1.0497	0.717

JMJD6	0.7792	0.4965	1.1251	0.8179
LDHA	1.5801	1.4743	1.4241	0.933
LGALS3	0.722	0.5471	0.8467	0.7474
LOX	0.8066	2.7132	0.8011	0.2813
MAP3K1	0.6329	0.5434	0.5664	0.5
MET	0.7579	0.5035	0.6783	0.4383
MIF	0.8409	1.2483	1.1019	1.6358
MMP9	0.7846	1.0644	1.0792	77.1717
MXI1	1.2311	1.057	10.4107	5.7358
NAMPT	0.8526	0.5704	1.0644	1.3755
NCOA1	0.6199	0.5548	0.6507	1.1728
NDRG1	7.2602	30.4844	12.8171	5.6569
NFKB1	0.669	0.5396	0.5625	0.4569
NOS3	0.6199	1.2746	0.7846	0.6598
ODC1	0.6598	0.3078	0.6242	1.1096
P4HA1	2.0139	3.4822	0.0001	1.5157
P4HB	0.7792	0.6029	0.7474	0.7371
PDK1	4.0558	3.6808	2.2658	2.8879
PER1	1.2483	0.8766	0.79	0.8236
PFKFB3	2.0279	1.9532	6.9644	4.6589
PFKFB4	5.3517	3.0525	8.0556	5.4264
PFKL	1.021	1.5369	1.1567	1.8025
PFKP	1.0353	0.8766	1.7654	2.395
PGAM1	1.1329	1.0425	1.1019	0.9794
PGF	0.8351	3.3404	7.4643	0.5285
PGK1	1.3287	2.0139	1.9319	1.8025
PIM1	0.79	0.8888	0.6417	0.5359
PKM	0.8351	0.6598	0.8645	1.4948
PLAU	0.6736	0.9931	0.4506	0.0759
RBPJ	0.8123	0.6926	0.9266	0.9266
RUVBL2	0.6552	0.4323	0.8011	0.7022
SERPINE1	0.7738	1.1408	0.8888	0.1768
SLC16A3	1.1173	2.2974	3.4822	1.9404
SLC2A1	2.1886	1.815	3.2944	1.9935
SLC2A3	2.5491	1.7411	1.6818	0.8888
TFRC	0.2207	0.4698	0.2176	0.6878
TP53	1.2746	1.3472	0.5704	0.1456
TPI1	1.0281	1.3013	1.0943	1.2142
TXNIP	0.2698	3.7064	0.5	0.9266
USF2	0.7631	0.5987	0.732	0.4538
VDAC1	0.8409	0.5548	1.0425	0.5322
VEGFA	4.084	4.2281	8.3977	5.1337
