# Supplementary figures and images for: Investigating Glioblastoma Response to Hypoxia
Source: Biomedicines. 2020 Aug 27;8(9):310. doi: 10.3390/biomedicines8090310 (PMC7555589; doi:10.3390/biomedicines8090310)

Figure S1

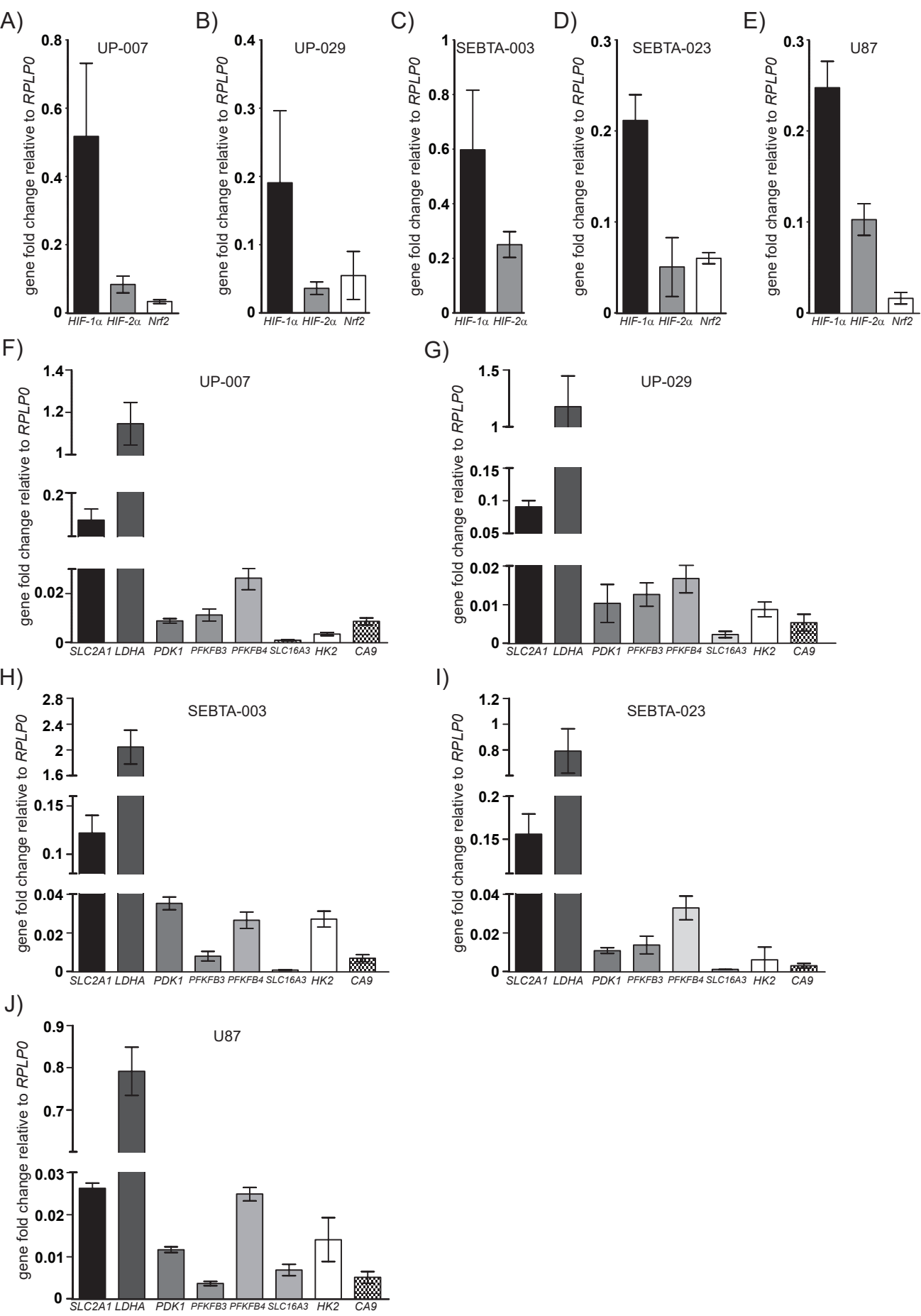

Supplement: Supplementary file 1 [file biomedicines-08-00310-s001.zip › Figure S1.pdf]

Figure S3

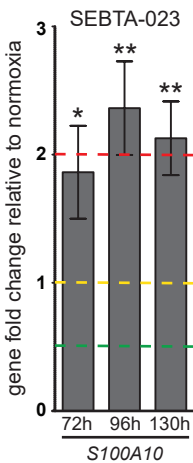

Supplement: Supplementary file 1 [file biomedicines-08-00310-s001.zip › Figure S3.pdf]

Figure S4

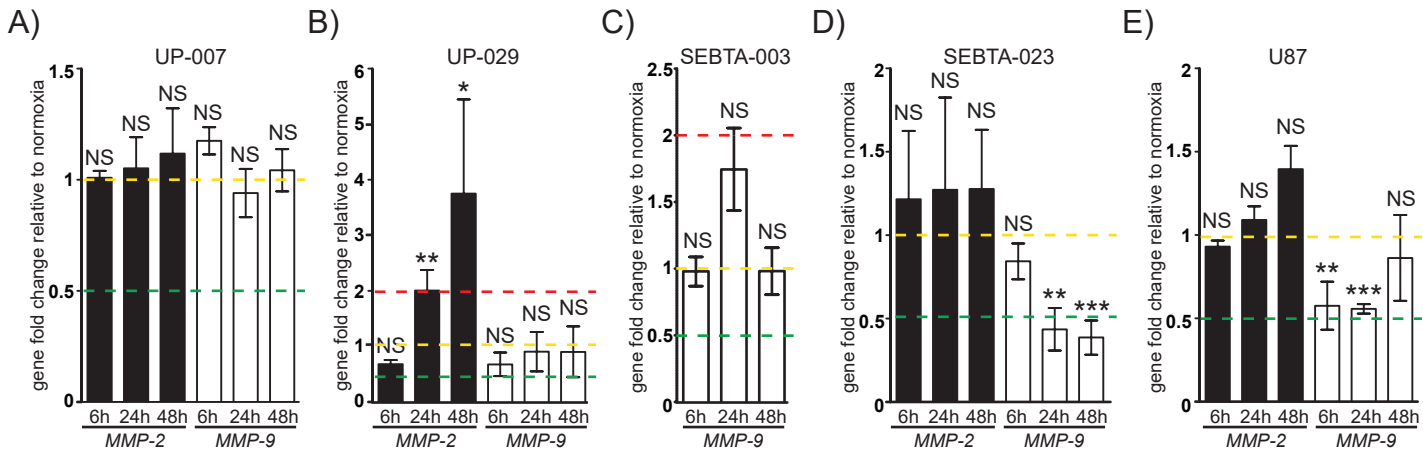

Supplement: Supplementary file 1 [file biomedicines-08-00310-s001.zip › Figure S4.pdf]

Figure S5

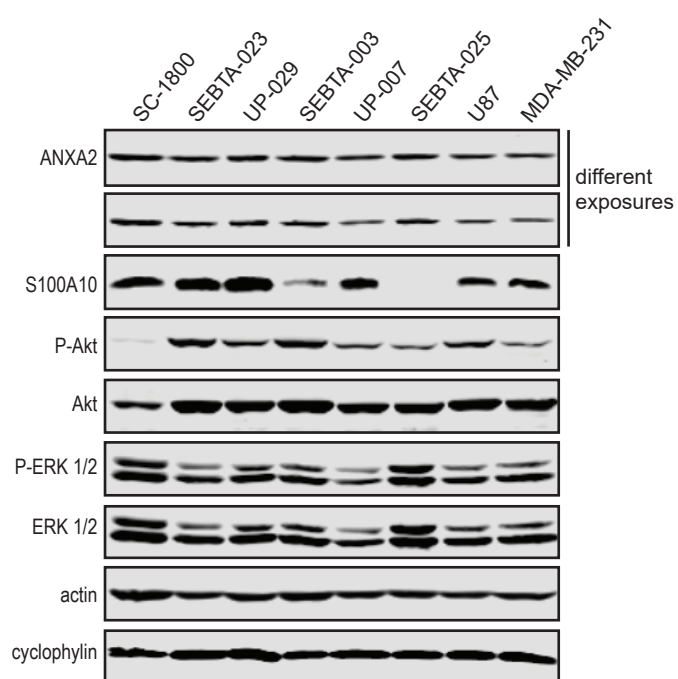

Supplement: Supplementary file 1 [file biomedicines-08-00310-s001.zip › Figure S5.pdf]

Figure S6

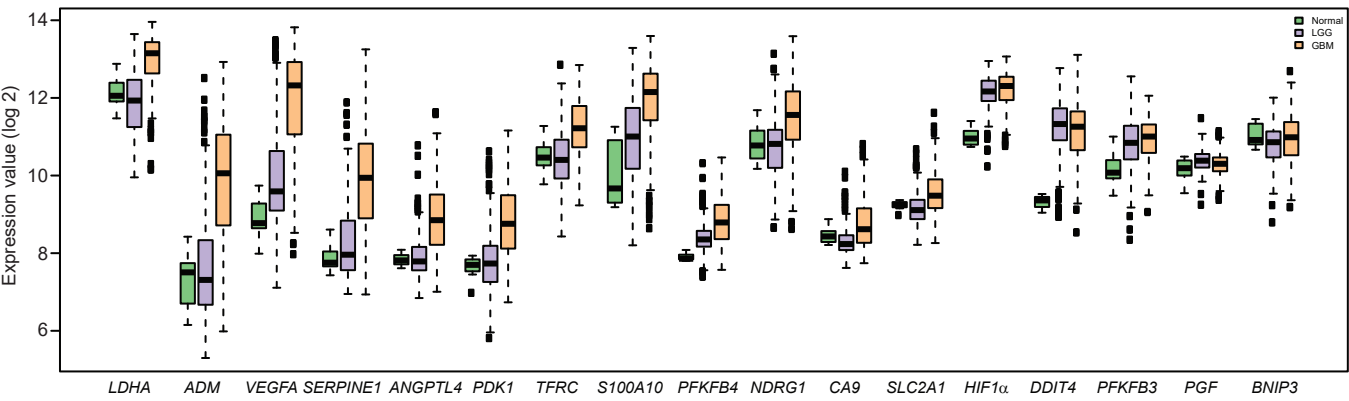

Supplement: Supplementary file 1 [file biomedicines-08-00310-s001.zip › Figure S6.pdf]

Figure S7

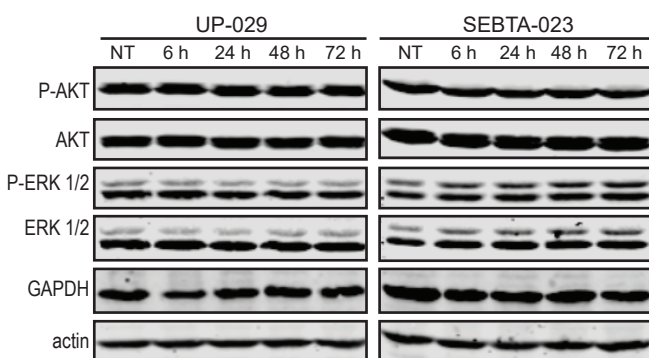

Supplement: Supplementary file 1 [file biomedicines-08-00310-s001.zip › Figure S7.pdf]
